# Synergistic photocatalytic aerobic oxidation of sulfides and amines on TiO_2_ under visible-light irradiation†
†Electronic supplementary information (ESI) available. See DOI: 10.1039/c4sc02891k



**DOI:** 10.1039/c4sc02891k

**Published:** 2014-10-30

**Authors:** Xianjun Lang, Wan Ru Leow, Jincai Zhao, Xiaodong Chen

**Affiliations:** a School of Materials Science and Engineering , Nanyang Technological University , 50 Nanyang Avenue , Singapore 639798 , Singapore . Email: chenxd@ntu.edu.sg; b Key Laboratory of Photochemistry , Beijing National Laboratory for Molecular Sciences , Institute of Chemistry , Chinese Academy of Sciences , Beijing 100190 , China . Email: jczhao@iccas.ac.cn

## Abstract

Selective photocatalytic aerobic oxidation, which can be conducted under ambient conditions, is of great importance towards achieving sustainable chemistry. However, its practical applications are undermined by several challenges, such as low selectivity, sluggish reaction rates, and the requirement of UV light irradiation. Herein, we report a new concept of synergistic photocatalytic oxidation, for which two seemingly irrelevant reactions can be achieved in one photocatalytic system through the synergistic interplay of reactants and catalyst. As proof of concept, two challenging reactions, the aerobic oxidation of sulfide and the aerobic oxidative formylation of amine with methanol, were employed to demonstrate such synergistic photocatalytic aerobic oxidation under visible-light irradiation. This work could pave the way for highly selective photoredox catalysis *via* rational design based on mechanistic insight.

## Abstract 2

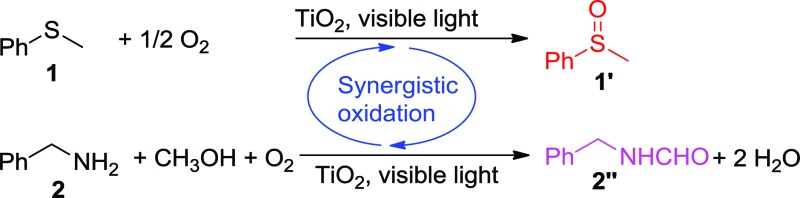
Visible-light-induced selective oxygenation of sulfides and oxidative formylation of amines in methanol with dioxygen were synergistically achieved on titanium dioxide.

## Introduction

The selective oxidation of organic compounds is one of the most vital transformations for upgrading raw starting materials into high-value-added products.^1–4^ However, it is also one of the most problematic chemical reactions, as stoichiometric amounts of toxic oxidants are traditionally required, leading to severe environmental impact and unsafe operational practices.^5–8^ O_2_, the most environmentally friendly and readily available oxidant, has been used to replace toxic oxidants, but its activation necessitates transition metal-based catalysts. The challenge is that most of such aerobic oxidation processes are carried out under harsh conditions such as high reaction temperature (>100 °C) and elevated O_2_ pressure (several MPa).^9–14^ Alternatively, photocatalysis can engender a paradigm shift by enabling the organic transformation to occur under very mild conditions.^15–23^ TiO_2_ is the most widely-used metal oxide for photocatalytic reactions, such as the selective oxidation of alkanes, alcohols and amines, *etc.*,^24–30^ but the large band gap (3.0–3.2 eV) only enables such reactions under UV light irradiation. In addition, the use of TiO_2_ is hindered by other challenges, such as low selectivity and a sluggish reaction rate.1
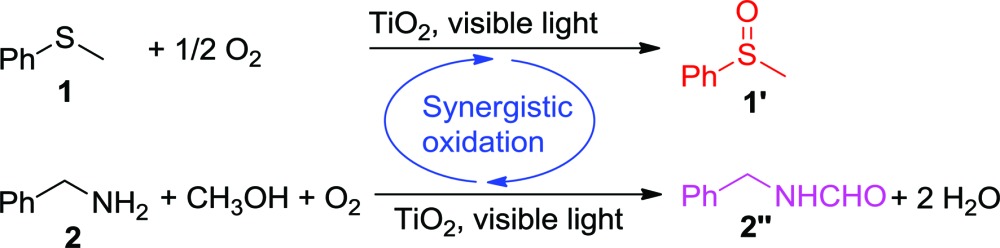

2
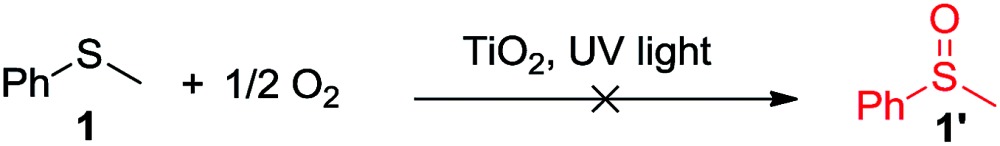

3
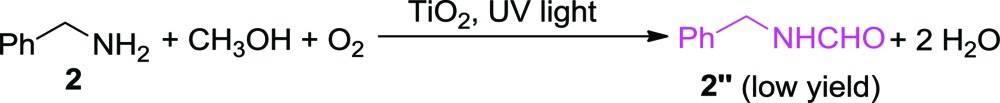



In this work, we report a new concept of synergistic photocatalytic oxidation, which can be carried out at an accelerated rate with a high selectivity of desired products under visible-light irradiation. Specifically, two seemingly irrelevant reactions can be achieved in one photocatalytic system through the synergistic interplay of reactants and catalyst. As proof of concept, we demonstrate that the synergistic aerobic oxidation of two substrates, sulfide **1** and amine **2**, occurs simultaneously on the surface of TiO_2_ under visible-light irradiation (eqn (1)). In contrast, attempts to perform these two reactions individually are not successful (eqn (2) and (3)). This new strategy brings about the high selectivity of two valuable products, with the solvent controlling the selectivity of one product (sulfoxide **1′**) and participating in the formation of another product (*N*-benzylformamide **2′′**). This finding provides a new perspective for the transformation of heteroatom-containing substrates by visible-light photocatalysis *via* a synergistic oxidation strategy.

## Results and discussion

**Fig. 1:**
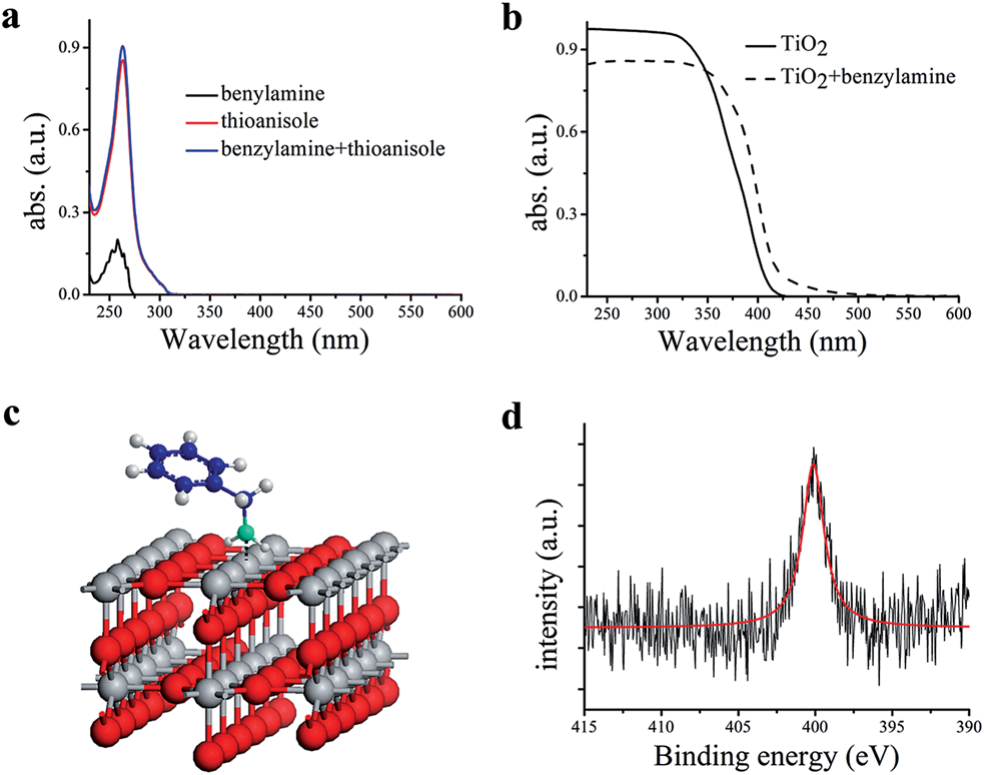
(a) UV-visible absorption spectra of thioanisole **1**, benzylamine **2** and their mixture in CH_3_OH; (b) UV-visible absorption spectra of TiO_2_ and after the adsorption of benzylamine **2** on TiO_2_; (c) the scheme of benzylamine **2** adsorption on rutile TiO_2_ (110), (the color convention is: O atom, red; Ti atom, gray; N atom, cyan; C atom, blue; H atom, white); (d), N1s XPS spectrum of TiO_2_ after benzylamine **2** adsorption, where the red line is used to guide the eye, a.u.: arbitrary unit.

In a typical experiment, commercially available Degussa P25 TiO_2_ was chosen as the model photocatalyst for the detailed investigation of the synergistic oxidation of two substrates. Degussa P25 TiO_2_ (hereafter denoted as TiO_2_ unless otherwise stated with the detailed characterization data presented in Fig. S1–S4†) is characterized by its ∼21 nm particle size and with a surface area of 52 m^2^ g^–1^. It comprises a mixture of anatase (75%) and rutile (25%) phases with the low energy {101} facet as the dominant facet. From the UV-visible spectrum (Fig. 1a), it can be seen that the absorptions of thioanisole **1**, benzylamine **2**, or a mixture of both are well below 325 nm, indicating that no reaction can occur from the direct photochemical activation of the substrates without the involvement of a photocatalyst. However, the formation of a visible-light-absorbing surface complex *via* the interaction of the substrate and TiO_2_ enables organic transformations under visible-light irradiation.^31,32^ When benzylamine **2** mixes with TiO_2_, a red shift of the absorption spectrum can be clearly observed (Fig. 1b), suggesting the formation of a surface complex through the adsorption of benzylamine **2** on TiO_2_. The surface complex (Fig. 1c) is akin to the donor–acceptor complex formed *via* the interaction of one reactant and the surface of TiO_2_,^33,34^ which enables the reaction to occur at a higher wavelength than that absorbed by individual substrates. Such a surface-complex was further evidenced by the observation of an N1s peak in the XPS spectrum (Fig. 1d), which was absent in the pure TiO_2_ sample (Fig. S3†).

Then, two challenging reactions, *i.e.* the aerobic oxidation of sulfide and the aerobic oxidative formylation of amine with methanol, were chosen to demonstrate the efficacy the synergistic photocatalytic oxidation. One reason for this is that the selective oxidation of sulfides to sulfoxides provides a very important intermediate for pharmaceuticals, but the tremendous challenge of controlling the product selectivity exists for heterogeneous photocatalytic systems utilizing O_2_ as the oxidant.^35–38^ In addition, the aerobic oxidative formylation of amine with methanol constitutes another important yet challenging reaction.^39–41^ This is because the formation of *N*-benzylformamide demands the prior selective oxidation of methanol to formaldehyde,^42–44^ which is a difficult reaction to realize, especially in the presence of a more fragile substrate, benzylamine **2**. Both of these oxidation reactions have never been successfully realized by TiO_2_ photocatalysis under UV irradiation (eqn (2) and (3)). However, it is interesting to note that aminocatalysis is very prevalent in synthetic chemistry.^45^ In particular, benzylic amines can function as organocatalysts in organic transformations.^46^ Therefore, we hypothesize that the aforementioned oxidation of benzylamines could possibly serve as the springboard in the pursuit of new and selective oxidation reactions.

As a control experiment, we first identified the low selectivity and efficiency of the two individual photo-oxidations of thioanisole **1** (Table S1†) and benzylamine **2** (Table S2†) catalyzed by TiO_2_ in the presence of O_2_. Only less than 10% of thioanisole **1** was transformed into the desired product sulfoxide **1′** with either the inert CH_3_CN or the protic CH_3_OH as the solvent. Moreover, the reaction ceased to proceed after 0.5 h, which might be due to the deactivation of TiO_2_ by thioanisole **1**. However, it is noted that the desired product could be obtained in quite high selectivity when CH_3_OH is used as the solvent (entry 2 and 4 of Table S1†). Meanwhile, the photo-oxidation of benzylamine **2** (entry 1 of Table S2†) resulted in the selective formation of the undesired product imine in the presence of CH_3_CN, and in the low selectivity of the desired product benzylformamide **2′′** in the presence of CH_3_OH.

**Fig. 2:**
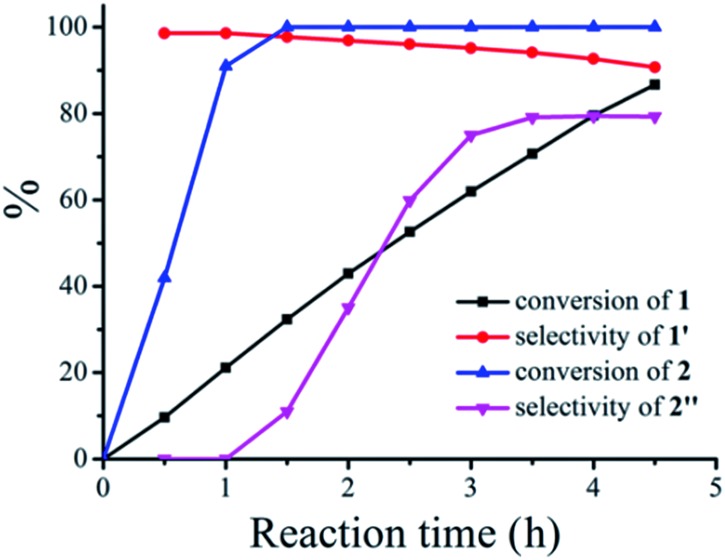
Reaction kinetics plot for the synergistic photocatalytic oxidation of thioanisole **1** and benzylamine **2** with O_2_ on TiO_2_ under visible-light irradiation. Reaction conditions: 0.3 mmol of thioanisole **1**, 0.1 mmol of benzylamine **2**, 40 mg of TiO_2_, 300 W Xe lamp, *λ* > 400 nm, 5 mL of CH_3_OH, 0.1 MPa of O_2_.

However, when these two seemingly irrelevant reactions are mixed in one photocatalytic system under visible-light irradiation using CH_3_OH as the solvent, the desired products can be clearly observed. The reaction kinetics for the conversion of the two substrates thioanisole **1** and benzylamine **2** with a ratio of 3 : 1 in CH_3_OH and the selective formation of their respective products is illustrated in Fig. 2. It can be observed that the steady conversion of thioanisole **1** to sulfoxide **1′** proceeds with high selectivity. The conversion of thioanisole **1** almost follows zero-order reaction kinetics with reaction constant of *k* = 12.45 mol L^–1^ h^–1^. The selectivity for **1′** decreases slightly with time, due to the minor over-oxidation of **1′** to sulfone which also follows zero-order reaction kinetics. In comparison with the conversion of thioanisole **1**, the conversion of benzylamine **2** was much more complicated and it includes an induction period (0–1 h) and a product formation period (1–4.5 h). Since CH_3_OH is a redox-active solvent, the selective aerobic oxidation of CH_3_OH to HCHO is achieved along with the formation of **1′**. HCHO then undergoes condensation with benzylamine **2** to form an intermediate. With the progress of time, the intermediate can be further transformed into *N*-benzylformamide **2′′**, an industrially important product. The corresponding reaction process for the plots is listed in Fig. S5.†

As a control experiment, when CH_3_OH is replaced by an inert organic solvent such as CH_3_CN, benzotrifluoride, ethyl acetate, or dichloromethane, the synergistic oxidation of thioanisole **1** and benzylamine **2** resulted only in the oxidation of benzylamine **2** to imine, with thioanisole **1** remaining almost intact in all cases (Table S3†). This suggests that an additional redox player is required to associate the two reactions. Here, we propose that the protic solvent CH_3_OH may act as the additional redox player required to enable the desired synergistic reaction due to the following reasons. Even though it is typically used as an h_vb_^+^ scavenger to quench other oxidation reactions in TiO_2_ photocatalysis. It is also a protic solvent, and protic solvent, H_2_O, has been reported to substantially increase the photocatalytic oxidation reaction rate.^47^ The selective oxidation of sulfides to sulfoxides indicated the need of protons in assisting the formation of products, and protic solvents might be helpful for the aerobic oxidation of sulfides under visible-light irradiation,^35–38^ even though they have rarely been used in TiO_2_ photocatalytic selective transformations.

It is of interest to note that the synergistic oxidation reaction is extremely robust. It is able to proceed under even milder conditions, such as with 0.1 MPa of air as the oxidant, albeit at a slightly slower reaction rate. The trend of the reaction with 0.1 MPa of air is the same as that for 0.1 MPa of O_2_ (Fig. S6†). This phenomenon was ascribed to the decrease in the amount of benzylamine **2** adsorbed on TiO_2_ in CH_3_OH compared with that in CH_3_CN (Table S7†). Thus, this finding might be limited as only a fraction of the entire visible-light range could be used.

**Scheme 1:**
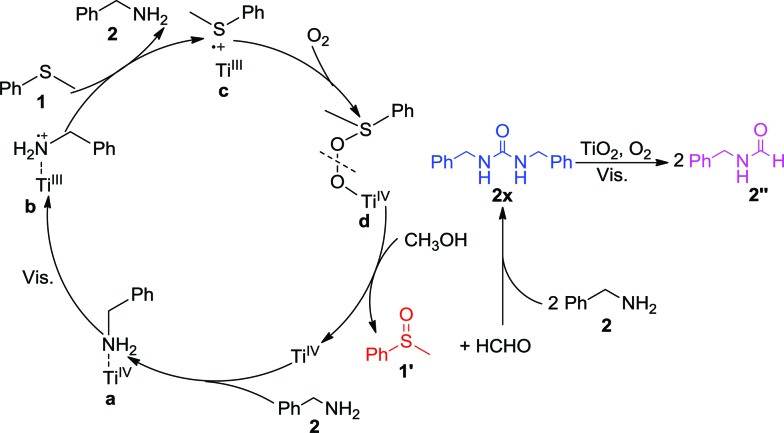
Proposed mechanism for the synergistic selective oxidation of thioanisole **1** and benzylamine **2** with O_2_ on TiO_2_ under visible-light irradiation.

Consequently, we propose the mechanism for the selective synergistic aerobic oxidation of thioanisole **1** and benzylamine **2** as shown in Scheme 1. The adsorption of benzylamine **2** on TiO_2_ leads to the formation of surface complex **a**, which shows activity under visible-light irradiation, thus facilitating electron transfer from the adsorbed benzylamine **2** to TiO_2_. This results in a positive charge at the surface-bound complex **b**, which could induce the oxidation of thioanisole **1** *via* single-electron transfer. As a result, the surface-bound S-centered positive free radical would be formed at the surface of TiO_2_ as complex **c**. During this stage, C(sp^3^)–S bond cleavage could occur with respect to the low selectivity to the desired product. This could be avoided by employing CH_3_OH instead of CH_3_CN as the solvent. The concerted incorporation of O-atoms into complex **c** forms complex **d**. The cleavage of **d** requires additional protons which could be provided by the solvent, CH_3_OH, in order to ensure the highly selective formation of **1′**. The protons from CH_3_OH could also prevent the oxidation products from blocking the reactive TiO_2_ surface, ensuring the sustainable oxidation of both substrates. The contribution of protons from alcohol have also been demonstrated in other oxygenation reactions in which free radical intermediates are involved. As the protons from CH_3_OH were consumed in the cleavage of **d**, HCHO was formed as the side product, which would in turn undergo condensation with benzylamine **2** to form intermediate **2x** which was confirmed by the GC-MS peak of *m*/*z* 242. As the disappearance of benzylamine **2** did not deter the selective formation of **1′**, this indicated that **2x** or other unknown species could also co-catalyze the oxidation of **1**. The nitrogen in the condensation product **2x** could coordinate with the Ti-atoms of TiO_2_ to initiate visible-light activity for the oxidation of thioanisole **1**, as well as its own decomposition to the final product **2′′** in the presence of HCHO. The transfer of an electron to O_2_ would restore the TiO_2_, thus completing the photocatalytic cycle. In this way, the selective synergistic aerobic oxidation of thioanisole **1** and benzylamine **2** to **1′** and **2′′** could be achieved.

To prove the above mechanism, we further studied the influence of solvent in this synergistic oxidation. Based on the prior observation that the proton-coupled electron transfer could control the reaction of free radicals, different protic solvents, *i.e.* different alcohols, were employed for the synergistic reactions. In all cases, the oxidation of thioanisole **1** to sulfoxide **1′** was unable to proceed under visible-light irradiation in the absence of benzylamine **2** (Table S4†), which was consistent with the reaction mechanism. However, when benzylamine **2** was added as the synergistic substrate, the oxidation of thioanisole **1** was significantly improved, with CH_3_OH delivering the best results due to it being the strongest organic protic solvent. Although benzylamine **2** was consumed in all three protic solvents, only CH_3_OH yielded the desired amide product, *N*-benzylformamide **2′′**, while isopropyl alcohol (IPA) and C_2_H_5_OH afforded imine as the product (Table S4†). From these results, it can be seen that the choice of solvent has an influential role on the mechanism of the reaction.

To better understand the essence of the synergistic effect, the amount of thioanisole **1** was fixed and the amount of benzylamine **2** varied, the results of which are summarized in Table 1. It can be seen that the formation of sulfoxide **1′** was almost the same for all ratios of thioanisole **1** to benzylamine **2**, except for the slight drop of conversion of thioanisole **1** with 0.1 equiv. of benzylamine **2** (entry 1, Table 1). This suggests that benzylamine **2** acts as an organocatalyst for the aerobic oxidation of thioanisole **1** on TiO_2_; the reaction could not proceed at all without benzylamine **2**. Although the conversion of benzylamine **2** was a very rapid process, able to achieve 100% conversion in merely 4 h or less, the formation of *N*-benzylformamide **2′′** from the resultant intermediate does not occur as rapidly. This leads to a difference in selectivity amongst entries 2–5 (Table 1), which could be enhanced by prolonging the reaction time. The ratio of 3 : 1 between thioanisole **1** and benzylamine **2** furnished good selectivities for both of the desired products at good conversions for both substrates. These results are in good agreement with the kinetics study in Fig. 2 and the proposed mechanism in Scheme 1.

**Table 1:** Influence of the ratio of substrates on the synergistic photocatalytic oxidation of thioanisole **1** and benzylamine **2** ^*a*^

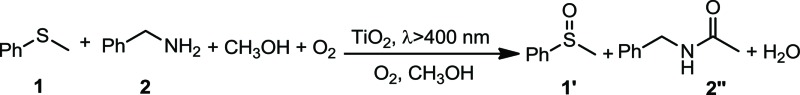						
Entry	Benzylamine (mmol)	Ratio	Thioanisole		Benzylamine	
			Con1^*b*^ (mol%)	Sel1^*b*^ (mol%)	Con2^*c*^ (mol%)	Sel2^*c*^ (mol%)
1	0.05	10 : 1	41	97	100	72
2	0.10	10 : 2	51	96	100	81
3	0.15	10 : 3	57	96	100	77
4	0.20	10 : 4	56	95	100	46
5	0.25	10 : 5	51	96	100	14

^*a*^Reaction conditions: 0.5 mmol of **1**, 0.1 MPa of O_2_, 40 mg of TiO_2_, 300 W Xe lamp, *λ* > 400 nm, 5 mL of CH_3_OH, 4 h.

^*b*^Determined by GC using chlorobenzene as the internal standard, conversion of **1**, selectivity of **1′**.

^*c*^Determined by GC using chlorobenzene as the internal standard, conversion of **2**, and selectivity of **2′′**.

In addition, it is noted that the scale-up of the synergistic reaction could be highly valuable. Thus, a study was conducted on the effect of substrate concentrations on the outcome of the synergistic reactions while maintaining the optimal ratio of 3 : 1 between the two substrates. It was discovered that, generally, the concentrations of both substrates could be simultaneously increased or decreased without a dramatic change in the conversions or selectivities of both **1′** and **2′′** (Table S5†). However, it was also observed that an overly high concentration of benzylamine **2** could result in a low selectivity of **2′′** due to side reactions (Table S5†).

More rigorous control experiments were carried out to prove the essential photocatalytic aerobic nature of the synergistic reaction system (Table S6†). It was observed that the synergistic oxidation reaction ceased to proceed when a *λ* > 420 nm longpass filter was applied (entry 5, Table S6†), which is different from our previous report in which the selective aerobic oxidation of amines in CH_3_CN proceeded smoothly with visible-light irradiation of the same wavelength range.^32^ In this scenario, one might suspect that the results were caused by the direct irradiation of rutile TiO_2_ in the photocatalyst used. If the reaction results were caused by the directed irradiation of the photocatalyst, UV irradiation should lead to better results. But our control experiment (entry 1, Table S6†) does not support this hypothesis.

To understand the scope of reaction, different types of thioanisoles were employed in the reaction while keeping benzylamine **2** constant. Table 2 revealed that the oxidation of substituted thioanisoles **1b–k** to the corresponding sulfoxides **1b′–k′** proceeded smoothly with very high selectivities, and that the conversions varied only slightly with the different substituted groups (entries 2–8, Table 2). However, very strong electron-withdrawing groups such as NO_2_ may significantly reduce the conversion rate of sulfide **1i** to sulfoxide **1i′** (entry 9, Table 2), resulting in a much longer time needed to obtain a higher conversion of **1i**. Besides the aforementioned electronic effect, the NO_2_ groups of **1i** could easily be adsorbed on the surface of TiO_2_ which could partially block the adsorption of **2**, contributing to the observed sluggish reaction rate. This would also cause the oxidation of **2** to yield benzaldehyde **2y** as the final product, rather than the desired *N*-benzylformamide **2′**. Meanwhile, changing the methyl group of **1** to ethyl as in the sulfide **1j** would not greatly influence the reaction rate of sulfide **1j** to sulfoxide **1j′** (entry 10, Table 2), but a phenyl group as in **1k** would result in a lower reaction rate and selectivity (entry 11, Table 2) in the conversion to **1k′**. Nevertheless, the summation of our results showed that the reaction is applicable to a large scope of thioanisoles, generally producing high conversions and selectivities for the product of sulfoxides.

**Table 2:** The selective aerobic oxidation of sulfides and benzylamine on TiO_2_ in CH_3_OH under visible-light irradiation^*a*^

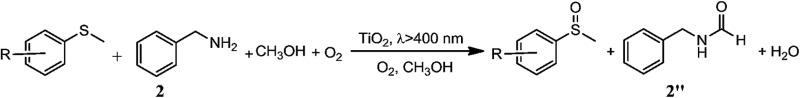						
Entry	Substrate (sulfide)	Product (sulfoxide)	Sulfide		Benzylamine	
			Con1^*b*^ (mol%)	Sel1^*b*^ (mol%)	Con2^*c*^ (mol%)	Sel2^*c*^ (mol%)
1	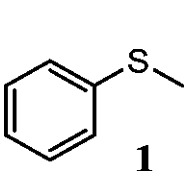	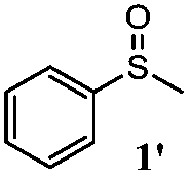	83	92	100	77
2	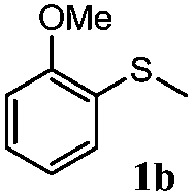	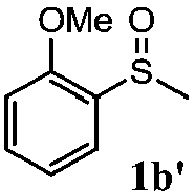	81	99	100	75
3	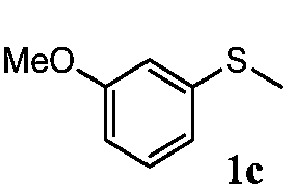	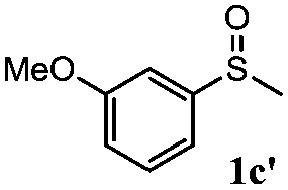	81	94	100	81
4	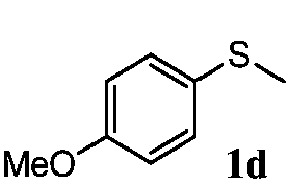	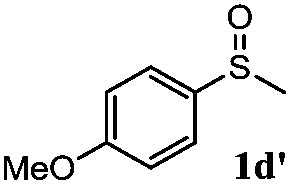	91	92	100	76
5	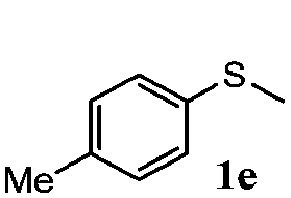	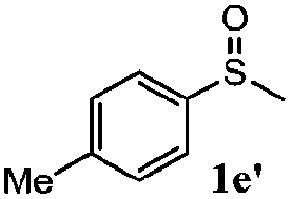	86	93	100	76
6	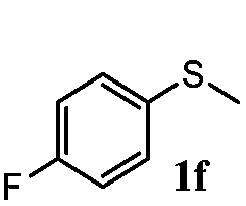	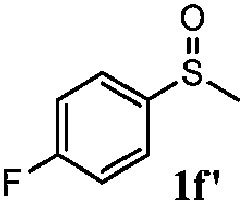	75	90	100	66
7	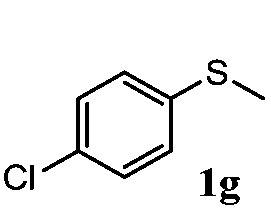	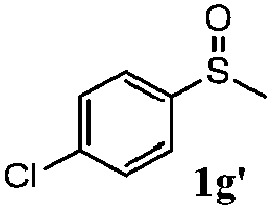	80	88	100	72
8	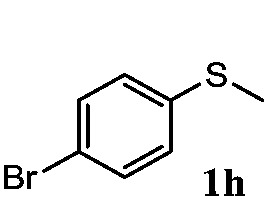	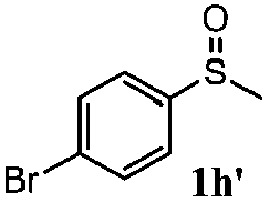	75	90	100	76
9	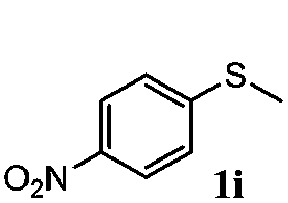	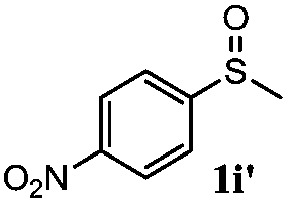	48	91	100	12^*d*^
10^*e*^	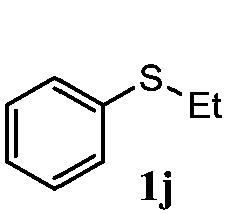	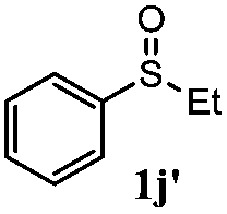	84	86	100	57
11	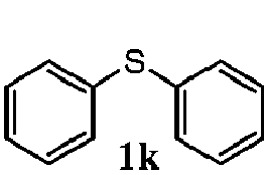	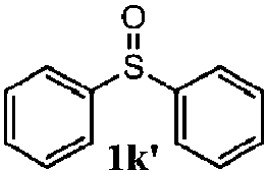	40	88	100	41

^*a*^Reaction conditions: 0.3 mmol of sulfide, 0.1 mmol of **2**, 0.1 MPa of O_2_, 40 mg TiO_2_, 300 W Xe lamp, 5 mL of CH_3_OH, *λ* > 400 nm, 4 h.

^*b*^Determined by GC using chlorobenzene as the internal standard, conversion of sulfide, selectivity of corresponding sulfoxide.

^*c*^Determined by GC using chlorobenzene as the internal standard, conversion of **2**, selectivity of **2′′**.

^*d*^Selectivity for benzaldehyde **2y**.

^*e*^0.1 mmol of 4-chlorobenzylamine. Me, methyl; Et, ethyl.

Finally, the scope of reaction with regard to benzylamines was investigated by testing different types of amines in the reaction while fixing the sulfide as thioanisole **1** (Table 3). It was observed that different primary benzylamines could yield high selectivities in the oxidation of thioanisole **1** to sulfoxide **1′** (entries 1–8, Table 3), but the conversion of thioanisole **1** for the substituted benzylamines is lower than that of benzylamine **2**. In addition, the selectivities for the corresponding *N*-benzylformamides of substituted benzylamines were all lower than that of benzylamine **2**. This is because, for the electron-donating groups, the oxidation of amines led instead to the formation of imine products, which could not induce the oxidation of thioanisole **1**, thus resulting in the lower conversion of thioanisole **1**. Meanwhile, for the electron-withdrawing groups, the slower reaction rates were due to the relative difficulty in donating the electron to the conduction band of TiO_2_, and could be compensated for by allowing longer reaction times. The selectivities for corresponding formamides could be improved with longer reaction times. It could also be seen that the existence of a heteroatom in the aromatic rings of the amines led only to a slight drop in the selectivity of formamides (entries 6–8, Table 3). For secondary benzylamines, no corresponding formamides were formed: benzaldehyde **2y** was the only product formed in the oxidation of secondary benzylamines **2j** and **2k** (entries 9 and 10, Table 3). This is in part because condensation between secondary benzylamines and HCHO to form the corresponding formamide is much more difficult than its primary counterpart. In addition, secondary benzylamines are not stable under the reaction conditions. The breakage of the C–N bond and ensuing oxidation led to the benzaldehyde **2y** as the main observed product.

**Table 3:** The selective aerobic oxidation of amines and thioanisole on TiO_2_ in CH_3_OH under visible-light irradiation^*a*^

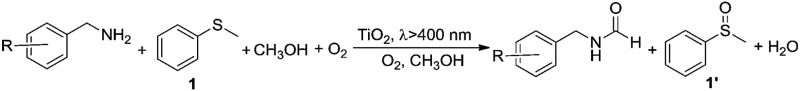						
Entry	Substrate (amine)	Product (formamide)	Amine		Thioanisole	
			Con2^*b*^ (mol%)	Sel2^*b*^ (mol%)	Con1^*c*^ (mol%)	Sel1^*c*^ (mol%)
1^*d*^	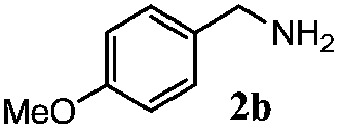	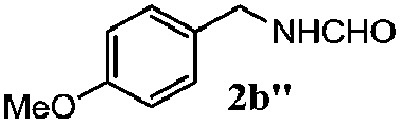	100	49	70	95
2	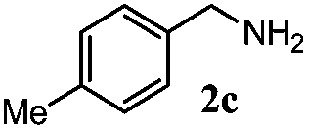	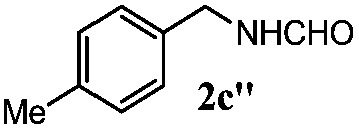	100	63	70	94
3	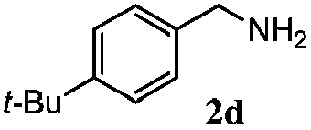	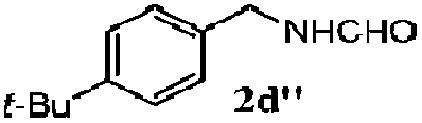	100	56	61	95
4	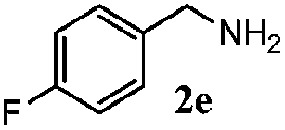	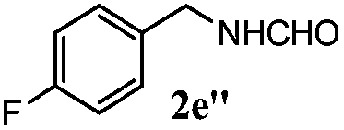	100	59	56	95
5	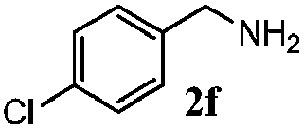	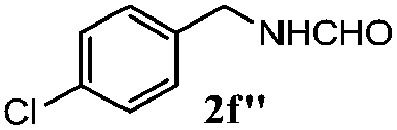	100	73	78	93
6	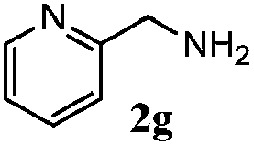	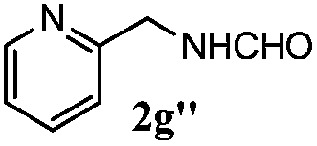	100	70	57	95
7	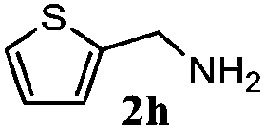	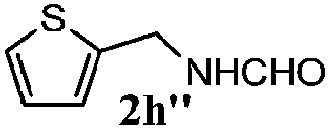	100	41	54	95
8	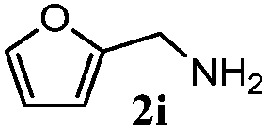	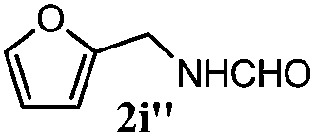	100	15	64	96
9	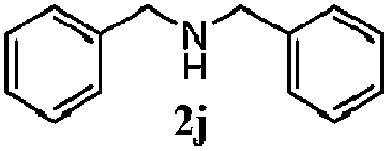	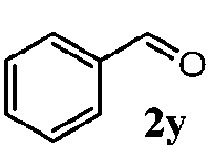	100	23	67	95
10	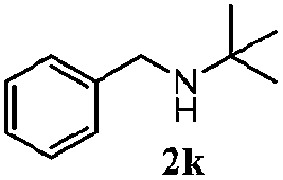	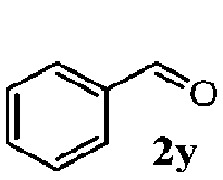	67	55	64	95

^*a*^Reaction conditions: 0.1 mmol of amine, 0.3 mmol of thioanisole **1**, 0.1 MPa of O_2_, 40 mg TiO_2_, 300 W Xe lamp, 5 mL of CH_3_OH, *λ* > 400 nm, 4 h.

^*b*^Determined by GC using chlorobenzene as the internal standard, conversion of amine, selectivity of corresponding formamide.

^*c*^Determined by GC using chlorobenzene as the internal standard, conversion of **1**; selectivity of **1′**.

^*d*^Imine (15%) as another product. Me, methyl; Et, ethyl; *t*-Bu, *tert*-butyl; MeO, methoxyl.

## Conclusions

To sum up, our novel concept of the synergistic aerobic photocatalytic reactions is extremely advantageous as it allows the following to be achieved: (1) a high selectivity for the two desired products; (2) reaction at high substrate concentrations; (3) clean reaction with O_2_ under visible-light irradiation; and (4) a long lifetime of the commercially available TiO_2_ photocatalyst. The present report represents a successful example of selective organic transformation with the TiO_2_ photocatalyst, which could pave the way for new discoveries of selective oxidation of organic compounds with an inexpensive metal oxide photocatalyst. Owing to inspiration from recent progress in both organocatalysis and techniques for the surface modification of semiconducting transition metal oxides, it is expected that more synergistic redox reactions can be achieved by judiciously selecting a pair of substrates and an appropriate solvent. In parallel, the functions of metal oxide nanomaterials are determined with specific surface area and texture,^48^ hierarchical structure^49^ and exposed crystal phase.^50^ The tunability of TiO_2_ in these aspects is one of the best amongst metal oxide materials. Thus, photocatalytic activity could also be improved to deliver better results.

## Experimental section

The reaction was irradiated using an Asahi Spectra MAX-303 300 W Xenon light source using a UV-VIS mirror model. In this mirror model, the irradiating wavelength range is 270–650 nm, thus the possible heating of the reaction medium by the infrared light is completely excluded. Additional Asahi Spectra longpass cutoff filters (>400 nm) are used to control the irradiation wavelength range during the reaction. The reaction medium was maintained at room temperature throughout the experimental process.

All of the reagents of the highest purity used were obtained from commercial suppliers and were used without further purification. In a typical reaction, 40 mg of TiO_2_, 0.3 mmol of thioanisole and 0.1 mmol of benzylamine were added to 5 mL of CH_3_OH in a Pyrex vessel. After the reaction mixture was stirred for 30 min in the dark to reach the adsorption equilibrium, O_2_ was purged into the Pyrex vessel to raise the initial pressure to 0.1 MPa. The reaction mixture was magnetically stirred at 800 rpm and illuminated with *λ* > 400 nm visible-light irradiation in an air-conditioned room to maintain the reaction temperature constantly at 25 °C.

At the end of reaction, the TiO_2_ photocatalyst particles were separated from the reaction mixture by filtration and the products were quantitatively analyzed by gas chromatography (GC) equipped with a flame ionization detector (FID) using chlorobenzene as the internal standard. The structures of products were confirmed by comparison of the retention times with standard samples and further confirmed by gas chromatography–mass spectrometry (GC–MS).

The quantitative measurements of conversions of the substrate and selectivities of products were made using a GC (Agilent 7890A) equipped with a flame ionization detector (FID) and Agilent Technology 19091J-413 capillary column (30 m × 0.32 mm × 0.25 μm) using high-purity N_2_ as the carrier gas. Standard analysis conditions: injector temperature 250 °C, detector temperature 300 °C, column temperature program: 50 °C (hold 1.5 min) raised up to 300 °C (hold 3 min) at a rate of 20 °C min^–1^. GC–MS analysis was performed on a Shimadzu GC 2010 gas chromatograph equipped with a Shimadzu GCMS-QP2010 Ultra mass spectrometer using a Restek (Rxi^®^-5Sil MS) capillary column (30 m × 0.25 mm × 0.25 μm), coupled with an electron ionization mass spectrometer with high-purity He as the carrier gas.

Full experimental details are provided in the ESI.†
