# Supplementary material for: Synergistic photocatalytic aerobic oxidation of sulfides and amines on TiO2 under visible-light irradiation
Source: Chem Sci. 2014 Oct 30;6(2):1075–82. doi: 10.1039/c4sc02891k (PMC5811138; doi:10.1039/c4sc02891k)
Supplement: Supplementary file 1 [file SC-006-C4SC02891K-s001.pdf]

Electronic Supplementary Material (ESI) for Chemical Science.
This journal is © The Royal Society of Chemistry 2014

Supporting Information:

Synergistic Photocatalytic Aerobic Oxidation of Sulfides and Amines on TiO_2 under Visible Light Irradiation

Xianjun Lang[1], Wan Ru Leow[1], Jincai Zhao,[2]* and Xiaodong Chen[1]*

1. School of Materials Science and Engineering, Nanyang Technological University, 50 Nanyang Avenue, Singapore 639798, Singapore *E-mail: chenxd@ntu.edu.sg

2. Key Laboratory of Photochemistry, Beijing National Laboratory for Molecular Sciences, Institute of Chemistry, Chinese Academy of Sciences, Beijing 100190, China *E-mail: jczhao@iccas.ac.cn

General:

The UV-visible absorption spectra of benzylamine, thioaniosole and the mixture of benzylamine and thioaniosole were recorded on a Shimadzu UV 2550 UV-vis Spectrophotometer. The detailed recording concentration: 2×10^{-3} M of benzylamine in MeOH; 2 $\times10^{-4}$ M of thioanisole in CH_3OH; a mixture of 6.7×10^{-4} M of benzylamine and 2 $\times10^{-4}$ M of thioanisole in CH_3OH.

The UV-visible absorption spectra of the solid samples were recorded on the same machine with a diffuse reflectance measurement accessory.

The phase composition of the Degussa P25 TiO_2 sample was identified by X-ray diffraction (XRD) using a Shimadzu 6000 X-ray diffractometer with Cu K_α radiation (λ = 1.541 78 Å).

The TEM image of Degussa P25 TiO_2 sample was recorded on a JEOL JEM-2010 transmission electron microscope (TEM) operating at 200 kV to obtain the detailed nanostructures.

X-ray Photoelectron Spectroscopy (XPS) were measured by a ESCALAB250XI. The incident radiation was Mg K_α X-ray (1253.6 eV) at 400 W and a charge neutralizer was turned on for acquisition. The binding energy of N1s was corrected by C 1s peak (284.8 eV) from residual carbon.

Table S1: Control experiments for the photocatalytic oxidation of thioanisole on TiO_2 in organic solvent[a]

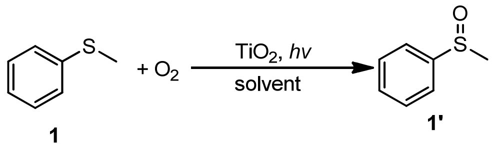

(1)

Entry	Solvent	Conditions	Conv. (mol%)[b]	Select. (mol%)[b]
**1**	CH_3CN	>350 nm	10	40
**2**	CH_3OH	>350 nm	5	95
**3**	CH_3CN	>400 nm	10	63
**4**	CH_3OH	>400 nm	6	93

Reaction conditions: 0.3 mmol of thioanisole, 0.1 MPa of O_2, 40 mg of TiO_2 (Degussa P25), 300 W Xe lamp, 5 mL of solvent, 3 h. Longpass cutoff filters are used to control the irradiation wavelength. [b] Determined by GC using chlorobenzene as the internal standard, conversion of thioanisole **1**, selectivity of methyl phenyl sulfoxide **1'**.

Table S2: Control experiment for the photocatalytic oxidation of benzylamine on Degussa P25 TiO_2 in organic solvent[a]

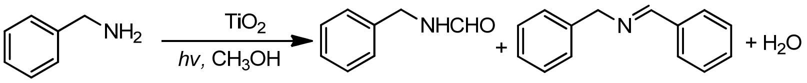

(2)

2 **2''** **2z**

Entry	Solvent	Conditions	Conv. (mol%)[b]	Select. (mol%)[b]
**1**	CH_3CN	>400 nm	100	85 (imine)
**2**	CH_3OH	>400 nm	100	38 (formamide)
**3**	CH_3OH	>350 nm	100	10 (formamide)

[a] Reaction conditions: 0.1 mmol of benzylamine, 0.1 MPa of O_2, 40 mg of Degussa P25 TiO_2, 5 mL of solvent, 3 h. [b] Determined by GC using chlorobenzene as the internal standard.

Table S3: The aerobic oxidation of thioanisole and benzylamine in inert organic solvent on TiO_2 under visible light irradiation[a]

Entry	Solvent	Conv1. (mol%)[b]	Select1. (mol%)[b]	Conv2. (mol%)[b]	Select2. (mol%)[b]
**1**	CH_3CN	10	63	--	--
**1'[c]**	CH_3CN	16	86	100	74
**2**	BTF	5	38	--	--
**2'[c]**	BTF	5	66	100	58
**3**	EtOAc	6	42	--	
**3'[c]**	EtOAC	12	80	100	75
**4**	DCM	4	65	--	--
**4'[c]**	DCM	8	90	100	58

[a] Reaction conditions: 0.3 mmol of thioanisole, 5 mL of solvent, 40 mg of Degussa P25 TiO_2, 300 W Xe lamp λ>400 nm, 0.1 MPa of O_2, 3 h, [b] Determined by GC using chlorobenzene as the internal standard. [c] 0.1 mmol of benzylamine was added. BTF, Benzotrifluoride; EtOAc, ethyl acetate; DCM, dichloromethane.

Table S4: The selective aerobic oxidation of thioanisole and benzylamine on TiO_2 in protic solvents under visible light irradiation[a]

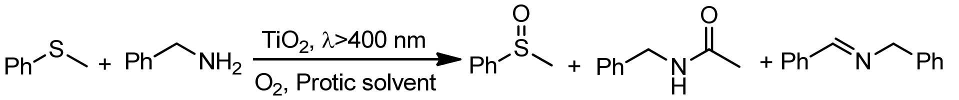

1 **2** **1'** **2''** **2c** (5)

Entry	Solvent	Thioanisole		Benzylamine	
		Conv1. (mol%)[b]	Select1. (mol%)[b]	Conv2. (mol%)[c]	Select2. (mol%)[c]
**1**	IPA	2	100	--	--
**1'[d]**	IPA	28	85	100	**2c** (64)[e]
**2**	C_2H_5OH	1	100	--	--
**2'[d]**	C_2H_5OH	45	88	100	**2c** (35)[e]
**3**	CH_3OH	6	93	--	--
**3'[d]**	CH_3OH	77	93	100	**2''** (79)[e]

[a] Reaction conditions: 0.3 mmol of **2**, 0.1 MPa of O_2, 40 mg Degussa P25 TiO_2, 300 W Xe lamp, 5 mL of solvent, λ>400 nm, 3 h. [b] Determined by GC using chlorobenzene as the internal standard, conversion of **2**, selectivity of **2b**. [c] Determined by GC using chlorobenzene as the internal standard, conversion of benzylamine. [d] 0.1 mmol of **1** was added. [e] Data in the parentheses is the selectivity for the indicated product for the oxidation of **1**, IPA, isopropanol.

Table S5: The scale-up for the synergistic photocatalytic oxidation of sulfide to amines [a]

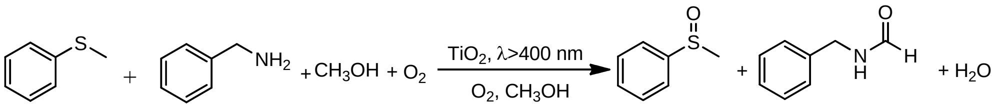

1 **2** **1'** **2''**

Entry	Substrates (mmol)	Time (h)	Conv1. (mol%)[b]	Select1. (mol%)[b]	Conv2. (mol%)[c]	Select2. (mol%)[c]
**1**	0.05+ 0.15	2	85	92	100	77
**2**	0.1+ 0.3	4	83	92	100	78
**3**	0.2+ 0.6	8	88	89	100	82
**4[d]**	0.3+ 0.9	12	83	91	100	42

[a] Reaction conditions: 5 mL of CH_3OH, 40 mg of TiO_2, 300 W Xe lamp, λ>400 nm, 0.1 MPa of O_2, [b] Determined by GC using chlorobenzene as the internal standard, conversion of thioanisole **1**, selectivity of sulfoxide **1'**; [c] Determined by GC using chlorobenzene as the internal standard, conversion of benzylamine **2**, selectivity of *N*-benzylformamide **2''**. [d] 15% of benzaldehyde **2y** and 5% of imine **2z** were the other products detected.

Table S6: Control experiment for the synergistic photocatalytic oxidation of thioanisole on Degussa P25 TiO_2 in organic solvent[a]

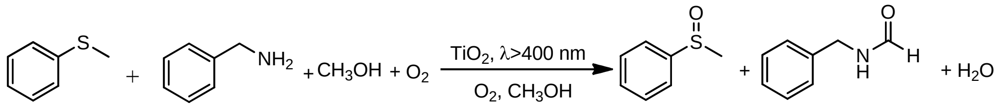

1 **2** **1'** **2''**

Entry	Conditions	Conv1. (mol%)[b]	Select1. (mol%)[b]	Conv2. (mol%)[c]	Select2. (mol%)[c]
**1**	>350 nm	30	92	100	10
**2**	>400 nm	83	92	100	78
**3[d]**	>400 nm	0	--	0	--
**4[e]**	>400 nm	0	--	0	--
**4[f]**	--	0	--	0	--
**5**	>420 nm	0	--	15	0

[a] Reaction conditions: 5 mL of methanol, 40 mg of Degussa P25 TiO_2, 300 W Xe lamp λ>400 nm, 0.1 MPa of O_2, 4 h. [b] Determined by GC using chlorobenzene as the internal standard, conversion of thioanisole, selectivity of sulfoxide; [c] Determined by GC using chlorobenzene as the internal standard, conversion of benzylamine, selectivity of *N*-benzylformamide. [d] Without O_2 with 0.1 MPa of N_2 as the atmosphere. [e] Without TiO_2. [f] Without λ>400 nm visible light irradiation.

Table S7: Adsorption difference of benzylamine on Degussa P25 TiO_2 in CH_3OH and CH_3CN

**Entry**	**Solvent**	**Adsorption amounts**
**1**	CH_3OH	3 μmol
**2**	CH_3CN	10 μmol

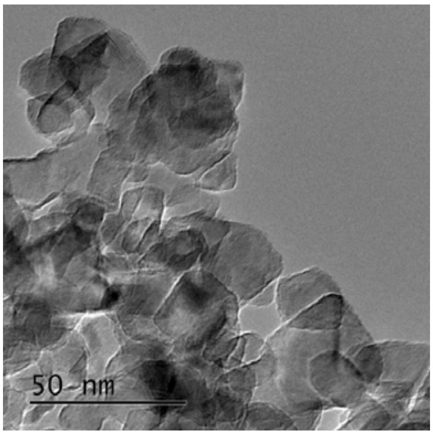

Figure S1: Transmission electron microscopy (TEM) images of Degussa P25 TiO_2

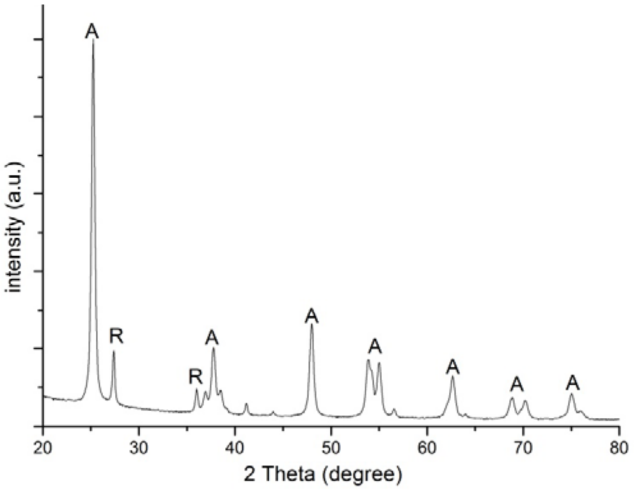

Figure S2: X-ray diffraction spectroscopy (XRD) of Degussa P25 TiO_2

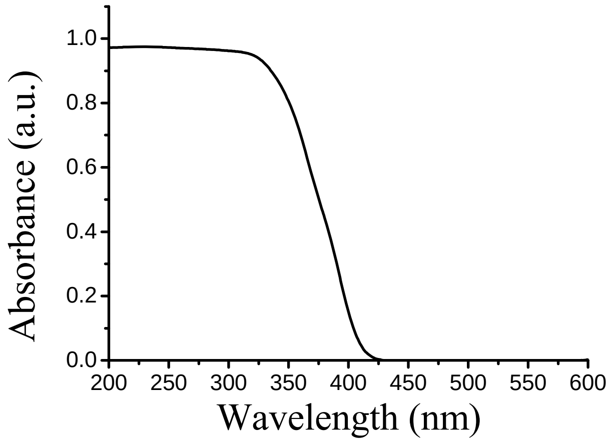

Figure S3: UV-visible absorbance spectroscopy of Degussa P25 TiO_2

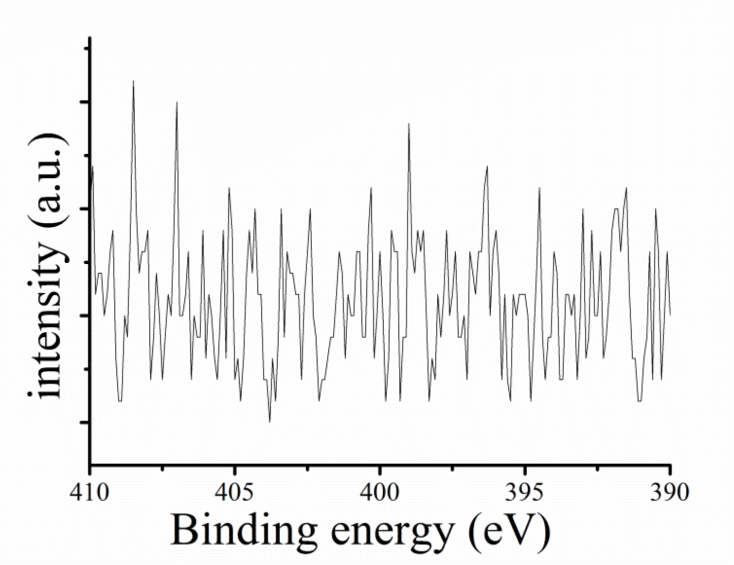

Figure S4: XPS spectroscopy of Degussa P25 TiO_2

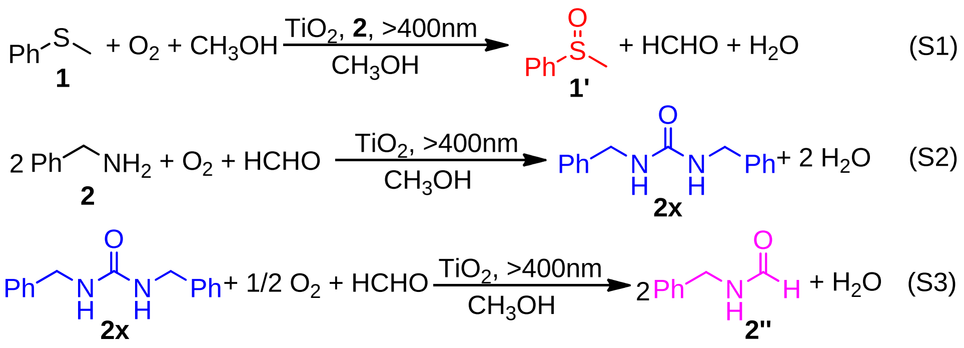

Figure S5: Products formation processes for the synergetic photocatalytic aerobic oxidation of sulfides and amines on TiO_2 under visible light irradiation

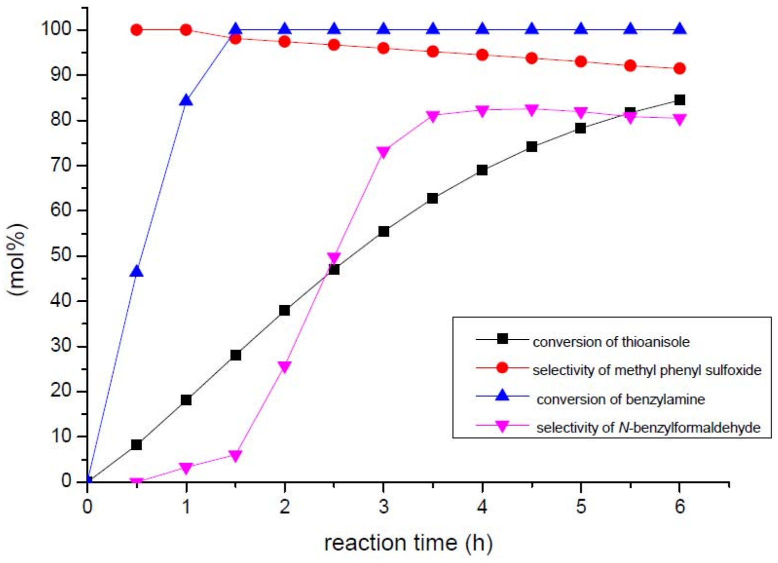

Figure S6: Reaction kinetic plot for the synergetic photocatalytic aerobic oxidation of sulfides and amines on TiO_2 under visible light irradiation. Reaction conditions: 0.3 mmol of thioanisole, 0.1 mmol of benzylamine, 40 mg of Degussa P25 TiO_2, 300 W Xe lamp, 5 mL of CH_3OH, λ>400 nm, 0.1 MPa of air

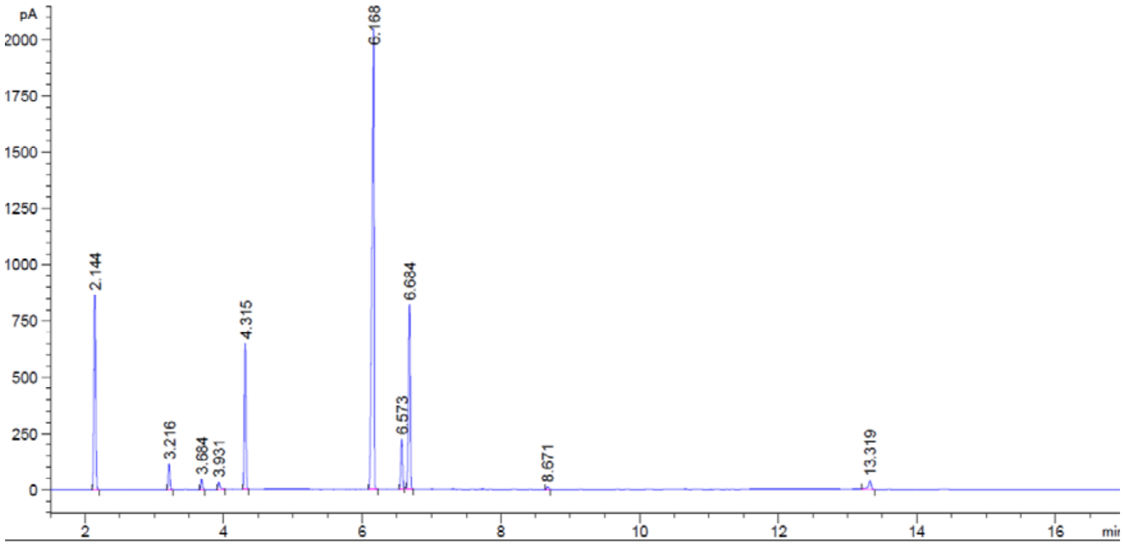

The GC-FID of Table 2 entry 1:

Retention time (min)	2.144	3.216	4.315	6.168	6.573	6.684	8.669
Chemical	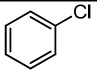	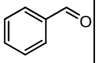	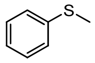	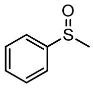	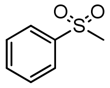	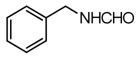	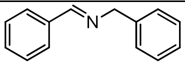

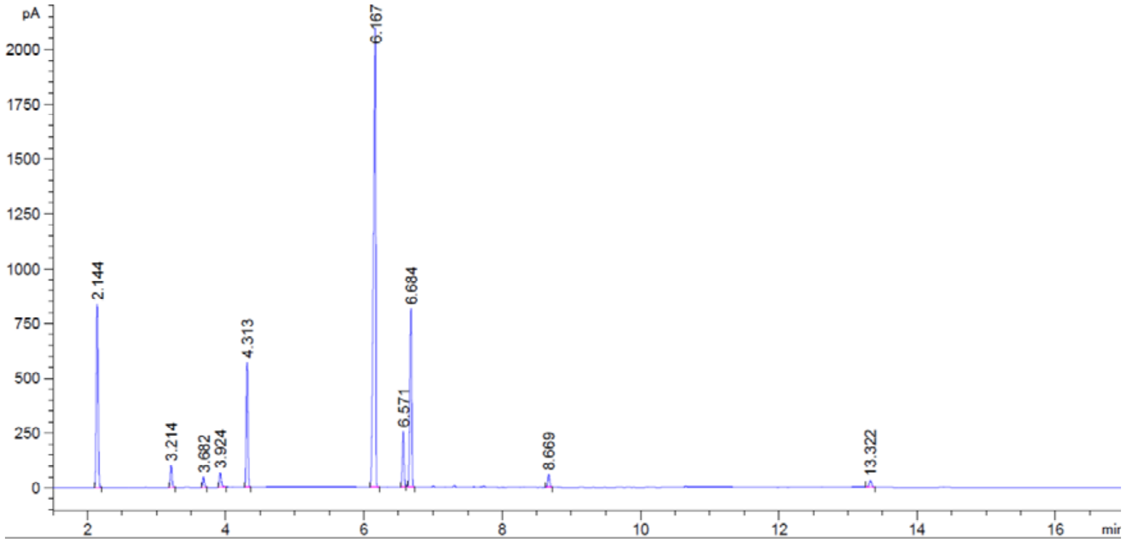

The GC-FID of Table 2 entry 2:

Retention time (min)	2.144	3.216	4.315	6.168	6.573	6.684	8.669
Chemical	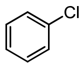	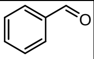	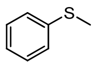	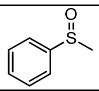	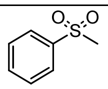	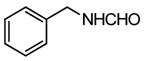	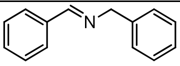

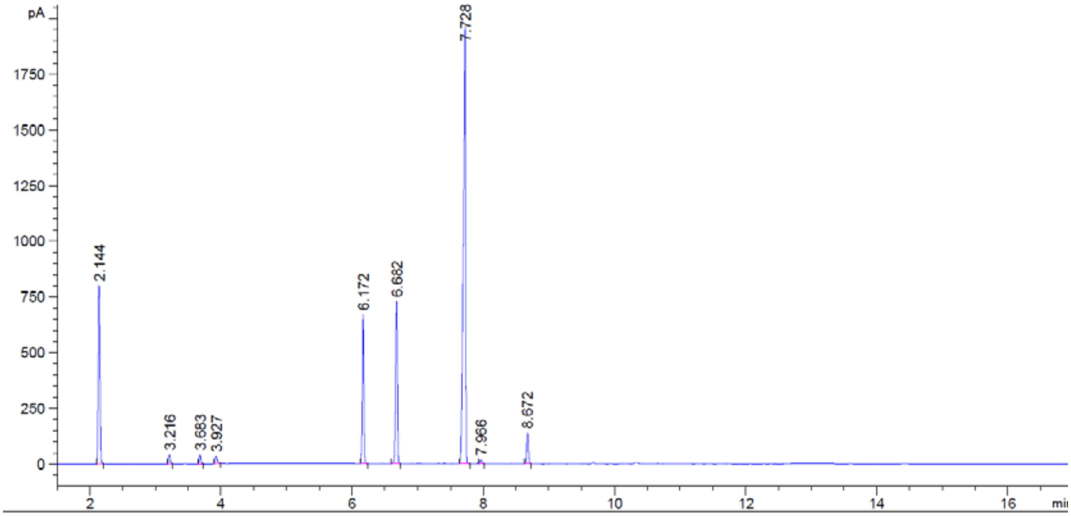

The GC-FID of Table 2 entry 3:

Retention time (min)	2.144	3.216	6.172	6.684	7.728	7.966	8.672
Chemical	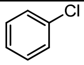	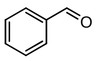	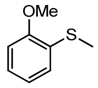	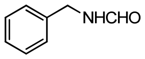	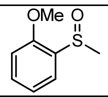	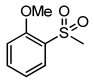	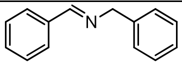

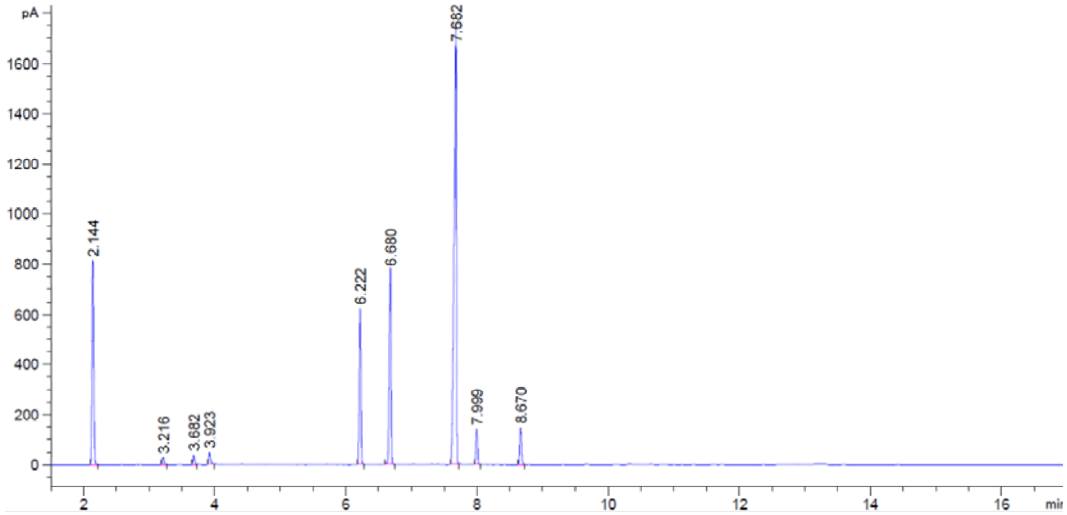

The GC-FID of Table 2 entry 4:

Retention time (min)	2.144	3.216	6.172	6.680	7.682	7.999	8.670
Chemical	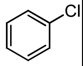	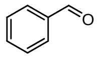	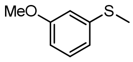	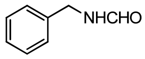	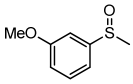	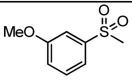	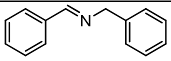

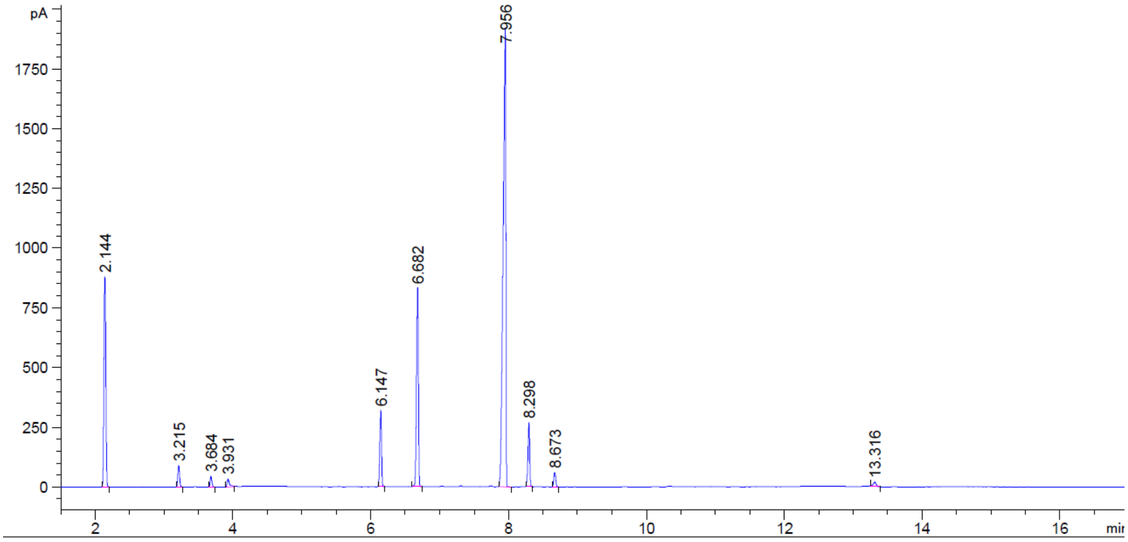

The GC-FID of Table 2 entry 5:

Retention time (min)	2.144	3.216	6.147	6.682	7.956	8.298	8.673
Chemical	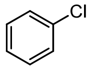	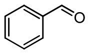	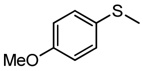	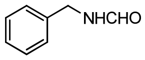	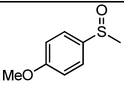	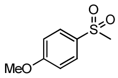	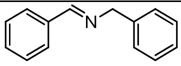

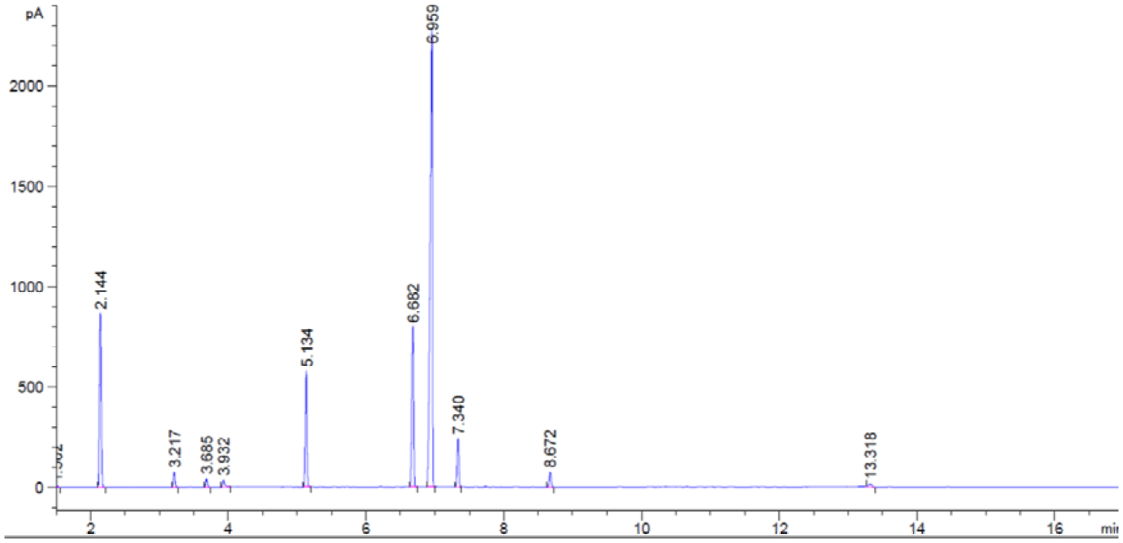

The GC-FID of Table 2 entry 5:

Retention time (min)	2.144	3.216	5.134	6.682	6.959	7.340	8.672
Chemical	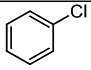	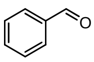	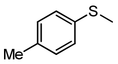	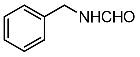	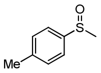	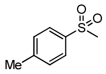	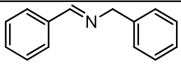

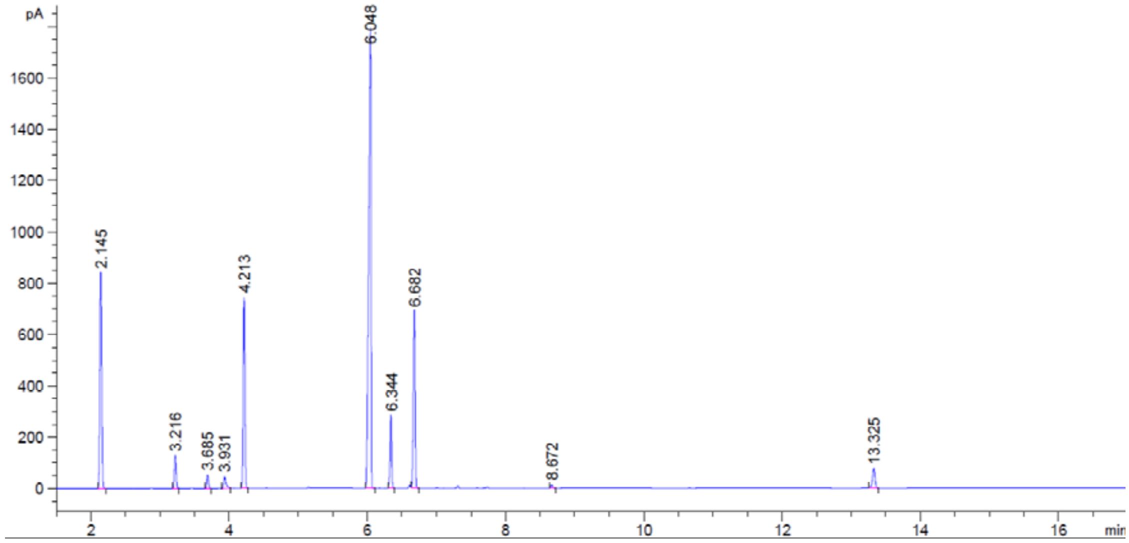

The GC-FID of Table 2 entry 6:

Retention time (min)	2.145	3.216	4.213	6.048	6.344	6.682	8.672
Chemical	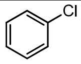	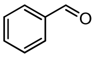	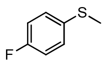	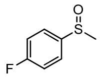	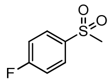	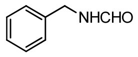	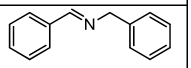

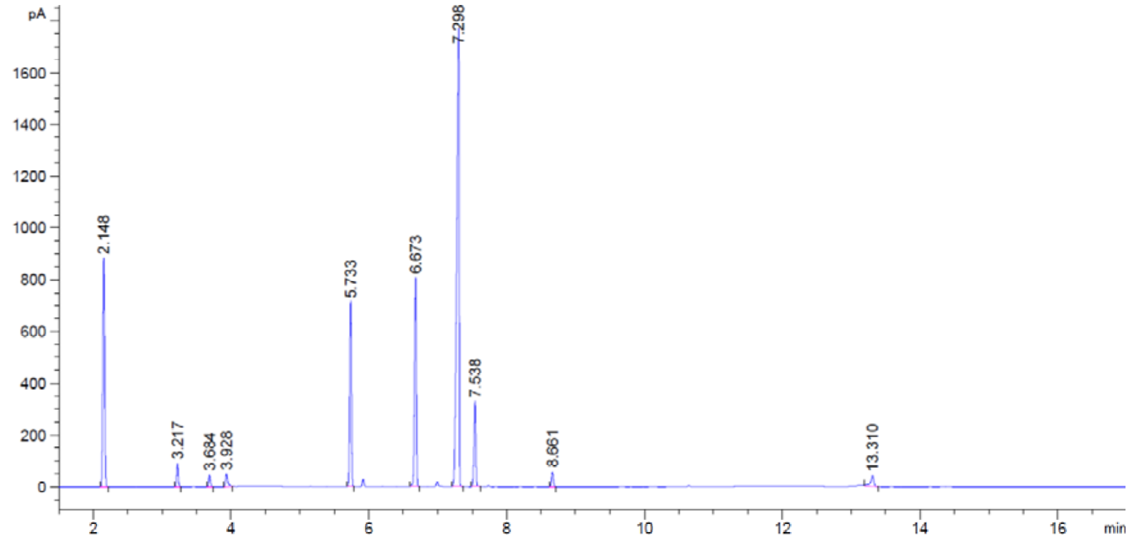

The GC-FID of Table 2 entry 7:

Retention time (min)	2.148	3.217	5.733	6.673	7.298	7.538	8.661
Chemical	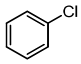	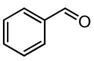	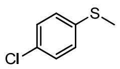	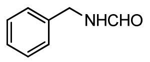	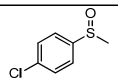	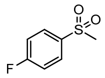	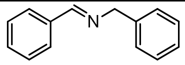

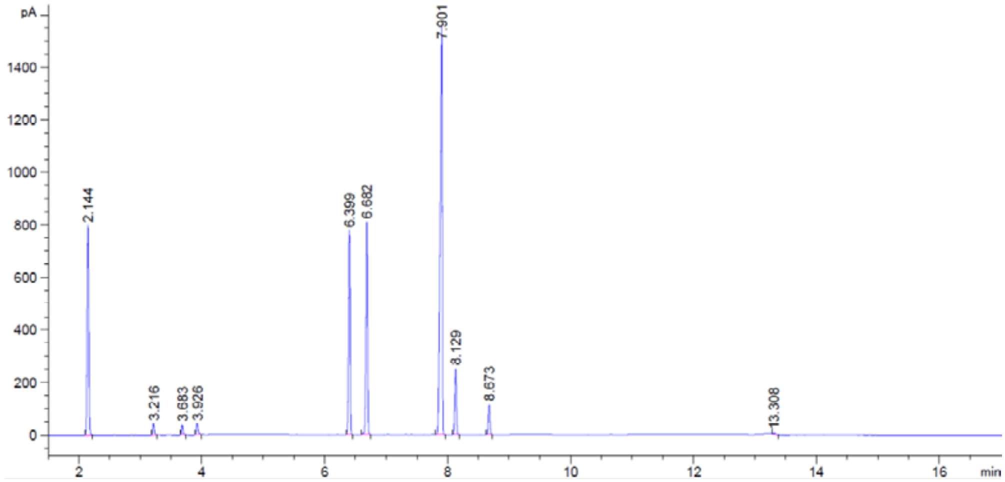

The GC-FID of Table 2 entry 8:

Retention time (min)	2.148	3.216	6.399	6.682	7.901	7.538	8.661
Chemical	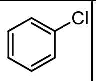	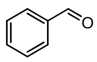	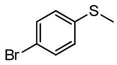	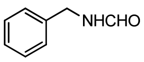	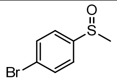	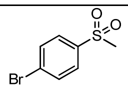	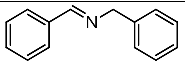

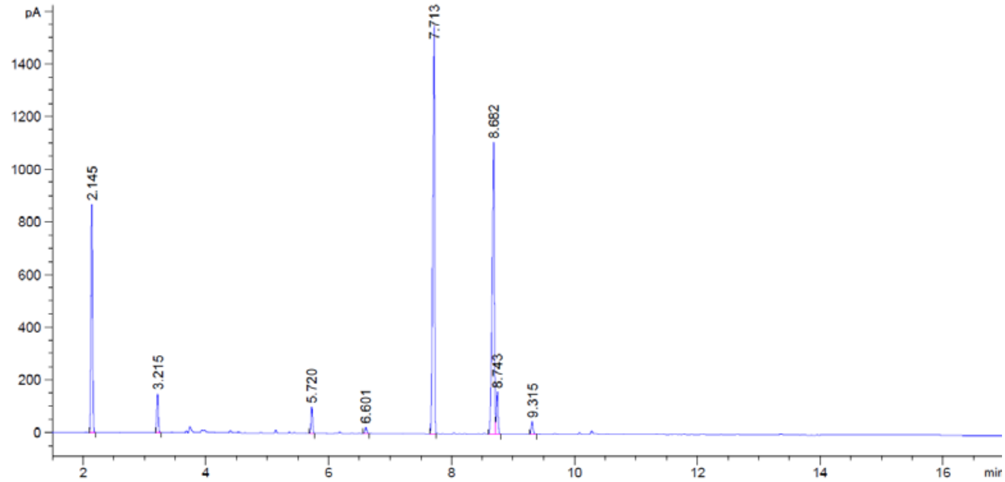

The GC-FID of Table 2 entry 9:

Retention time (min)	2.148	3.216	7.713	8.682	8.743
Chemical	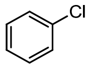	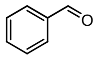	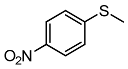	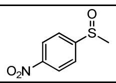	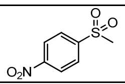

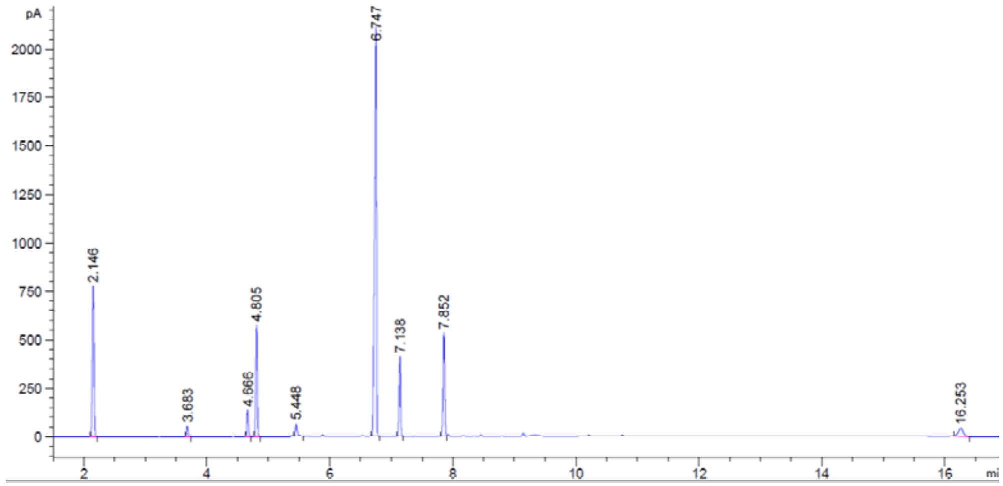

The GC-FID of Table 2 entry 10:

Retention time (min)	2.148	3.216	4.805	6.747	7.138	7.852
Chemical	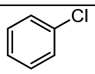	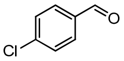	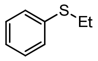	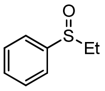	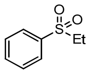	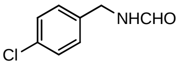

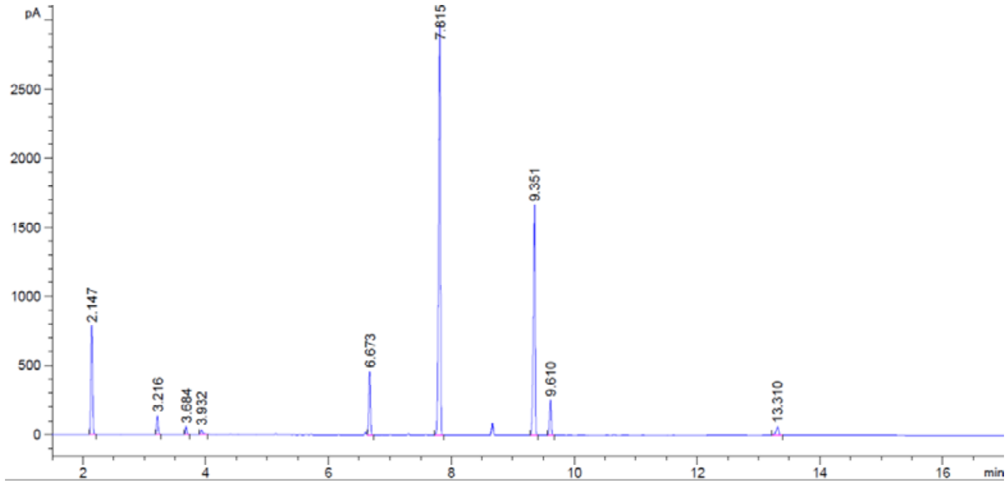

The GC-FID of Table 2 entry 11:

Retention time (min)	2.148	3.216	6.673	7.615	9.351	9.610
Chemical	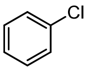	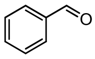	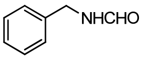	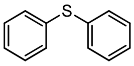	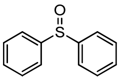	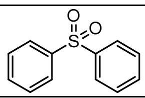

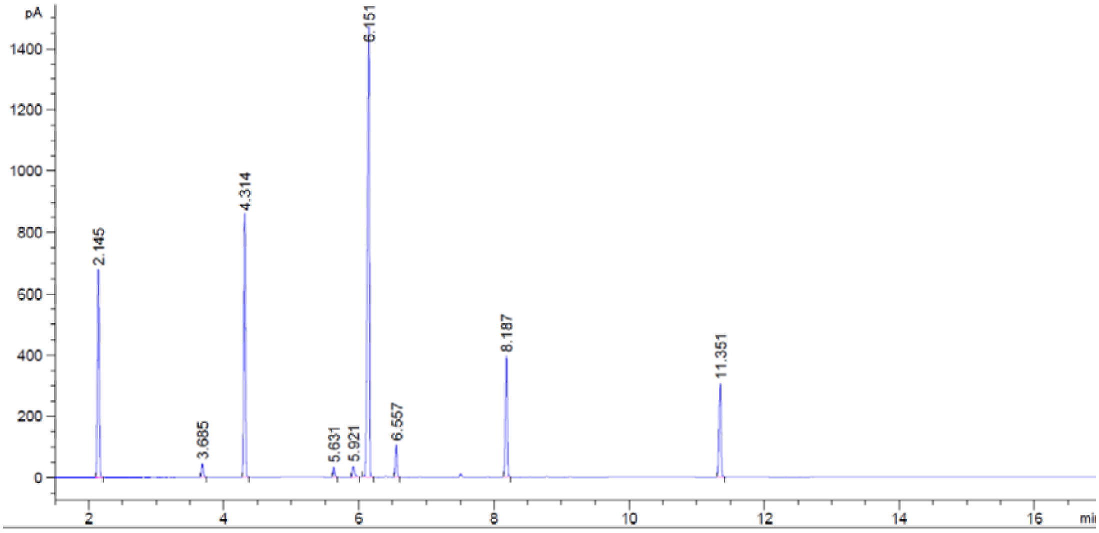

The GC-FID of Table 3 entry 1:

Retention time (min)	2.145	4.314	5.563	6.151	6.557	8.187	11.351
Chemical	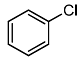	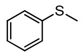	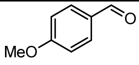	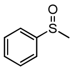	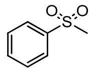	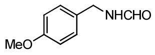	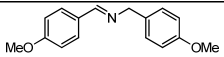

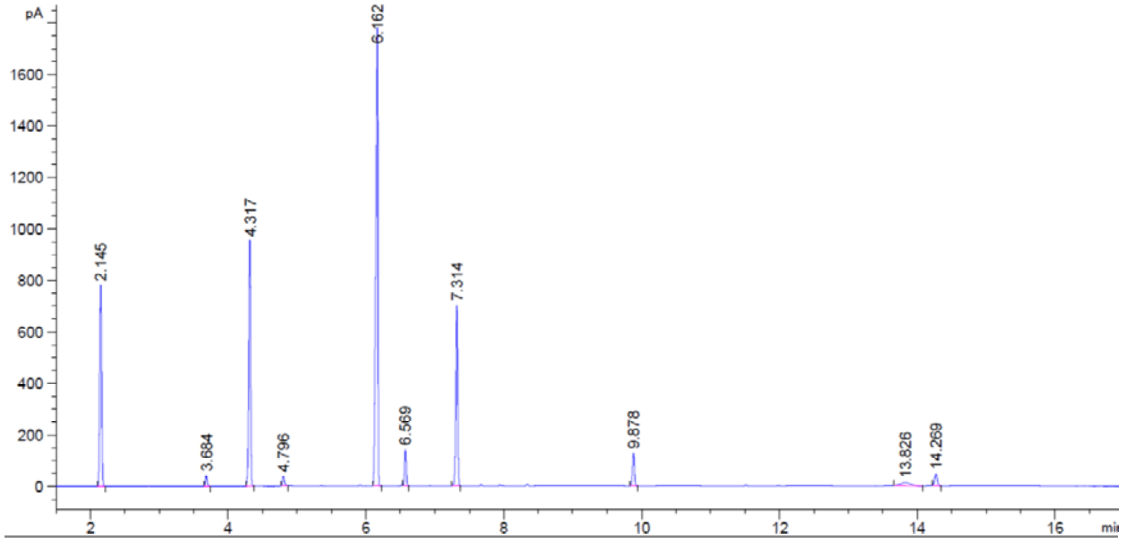

The GC-FID of Table 3 entry 2:

Retention time (min)	2.145	4.796	4.317	6.162	6.569	7.314	9.878
Chemical	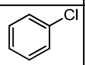	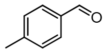	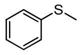	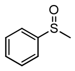	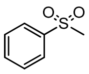	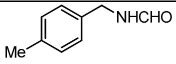	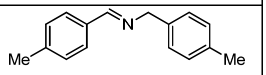

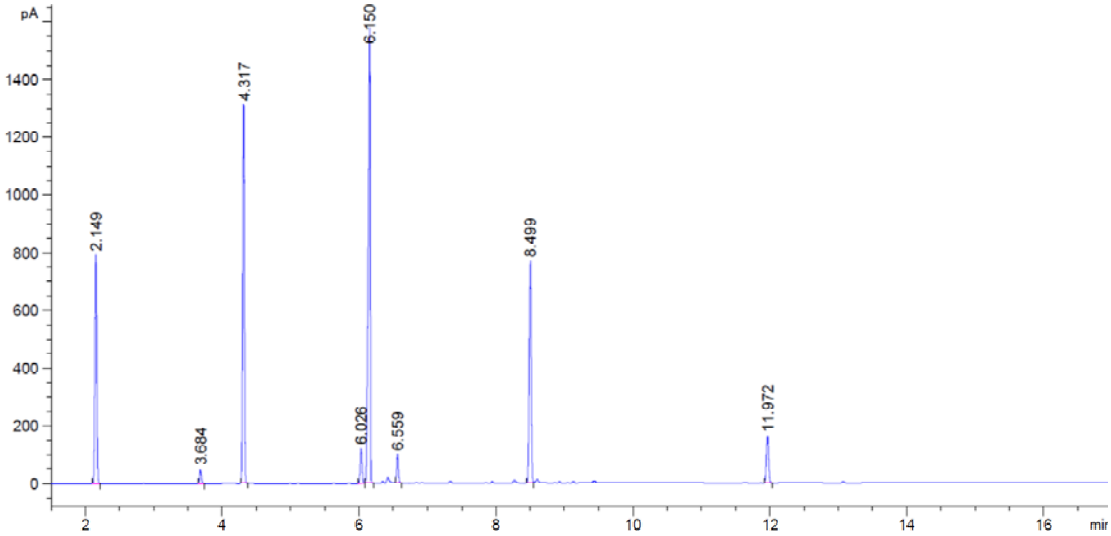

The GC-FID of Table 3 entry 3:

Retention time (min)	2.149	4.317	6.026	6.150	6.559	8.499	11.972
Chemical	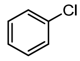	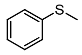	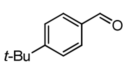	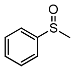	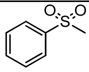	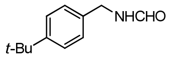	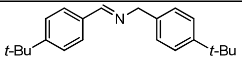

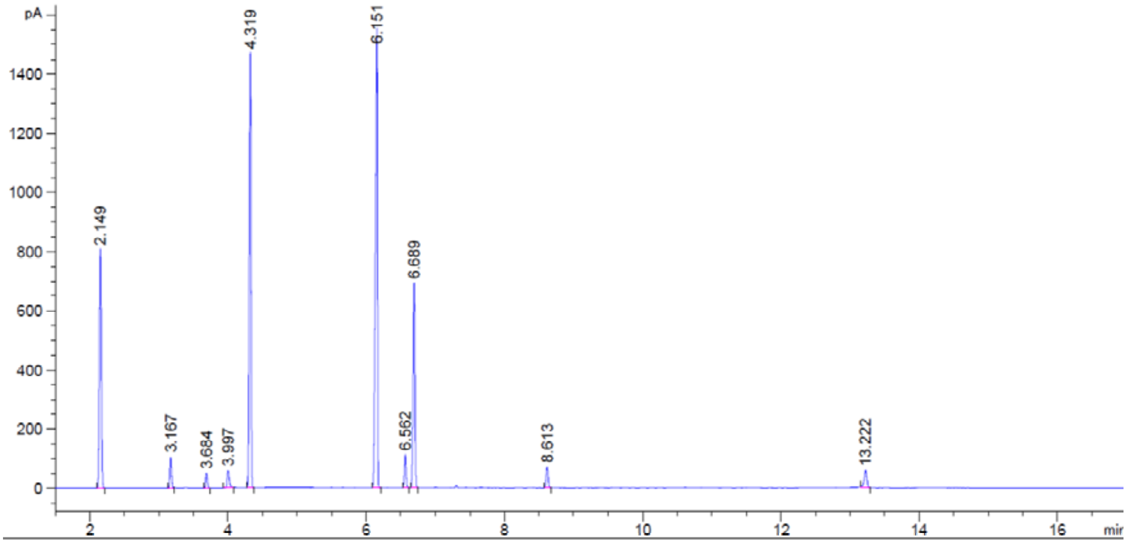

The GC-FID of Table 3 entry 4:

Retention time (min)	2.149	3.167	4.319	6.151	6.562	6.689	8.613
Chemical	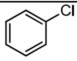	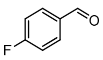	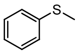	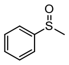	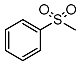	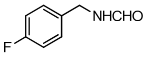	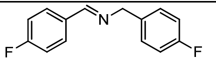

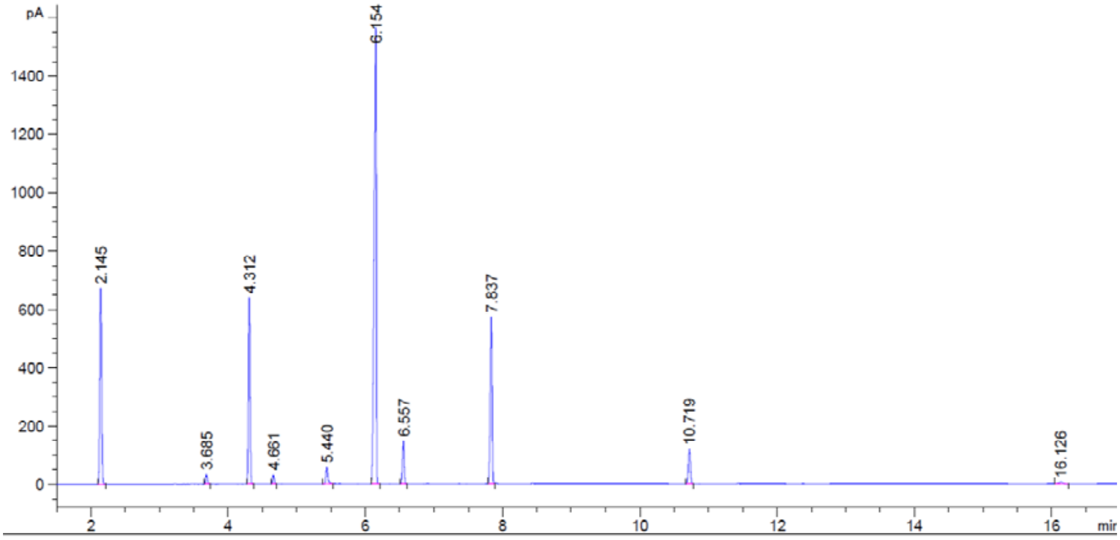

The GC-FID of Table 3 entry 5:

Retention time (min)	2.149	4.312	4.661	6.154	6.557	7.837	10.719
Chemical	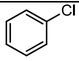	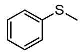	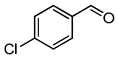	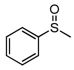	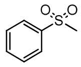	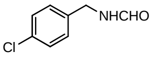	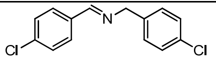

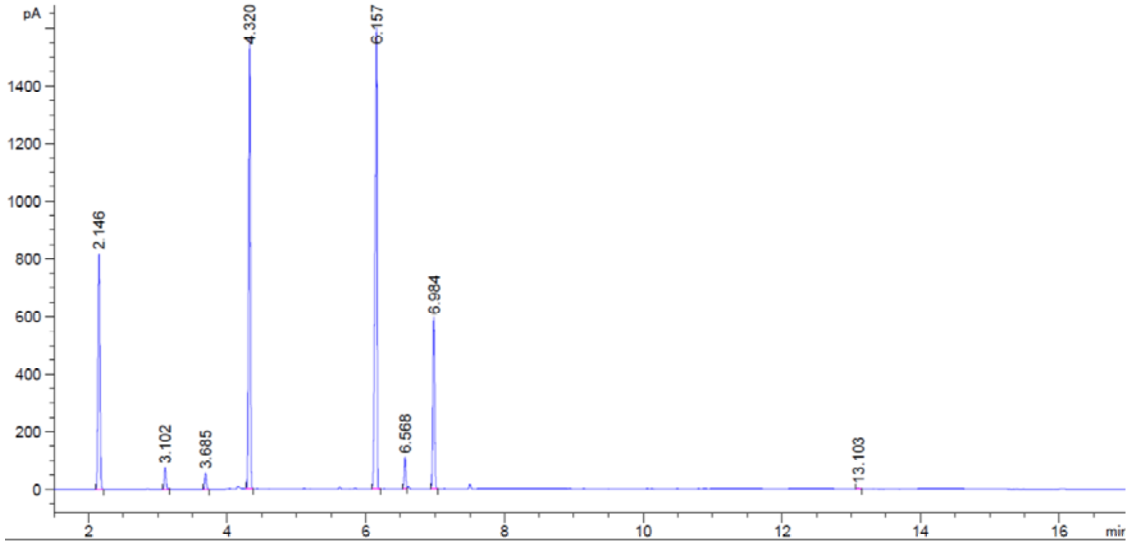

The GC-FID of Table 3 entry 6:

Retention time (min)	2.149	3.102	4.320	6.157	6.568	6.984
Chemical	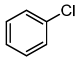	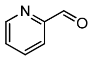	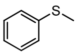	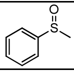	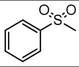	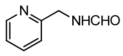

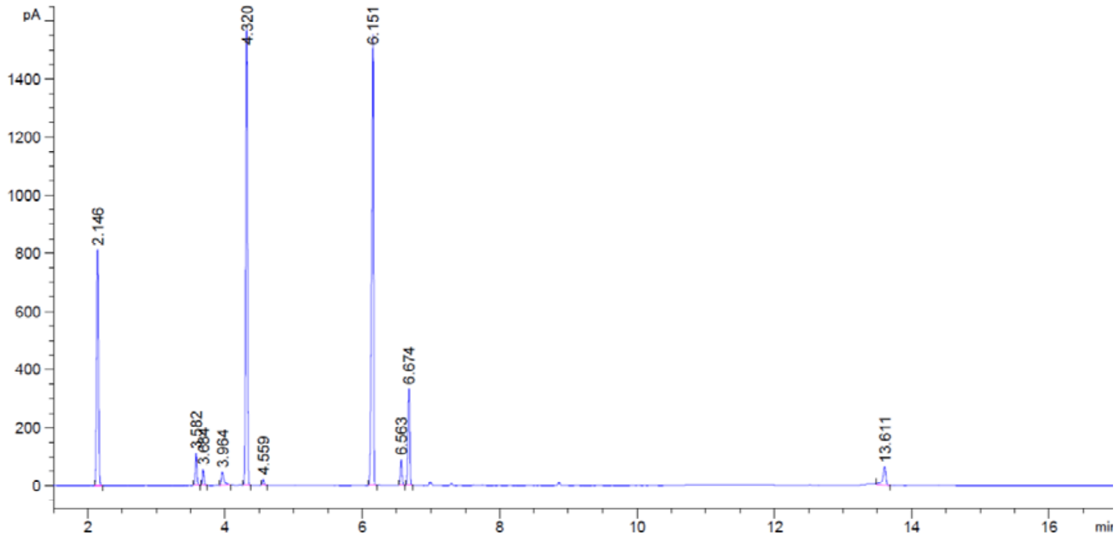

The GC-FID of Table 3 entry 7:

Retention time (min)	2.149	3.582	4.320	6.151	6.563	6.674
Chemical	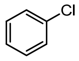	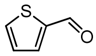	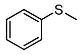	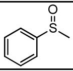	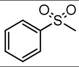	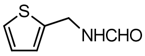

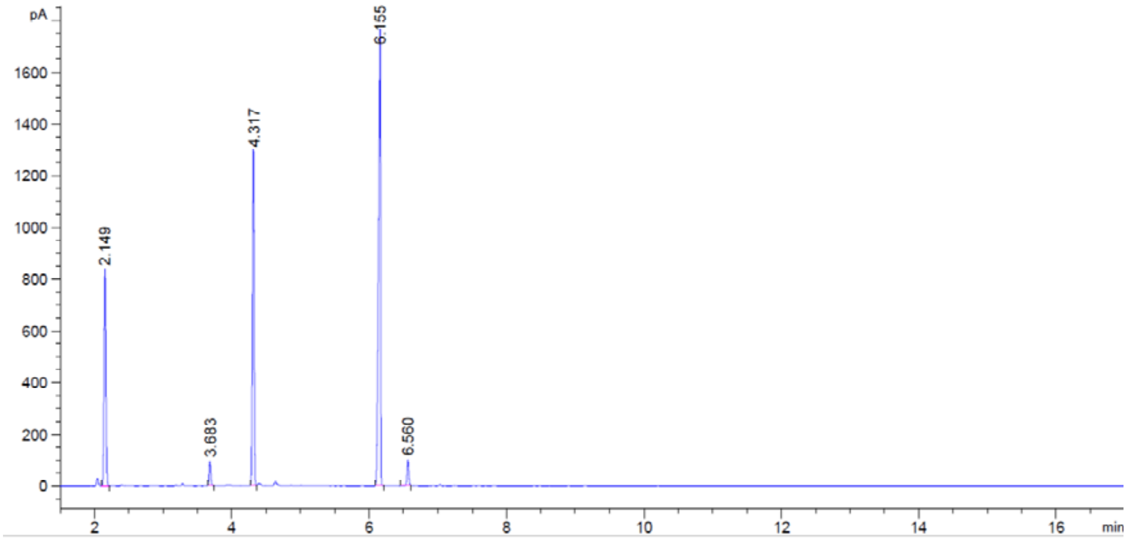

The GC-FID of Table 3 entry 8:

Retention time (min)	2.149	3.683	4.317	6.155	6.560
Chemical	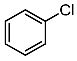	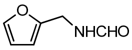	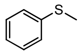	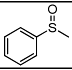	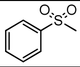

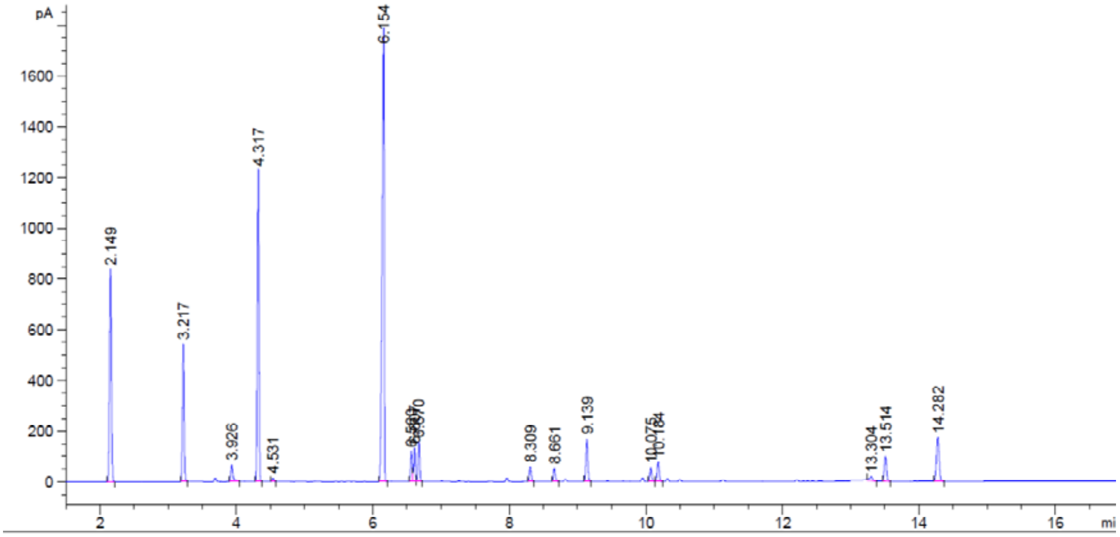

The GC-FID of Table 3 entry 9:

Retention time (min)	2.145	3.217	4.317	6.154	6.560	8.661
Chemical	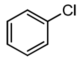	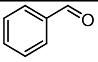	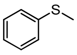	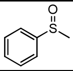	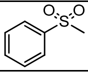	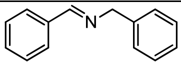

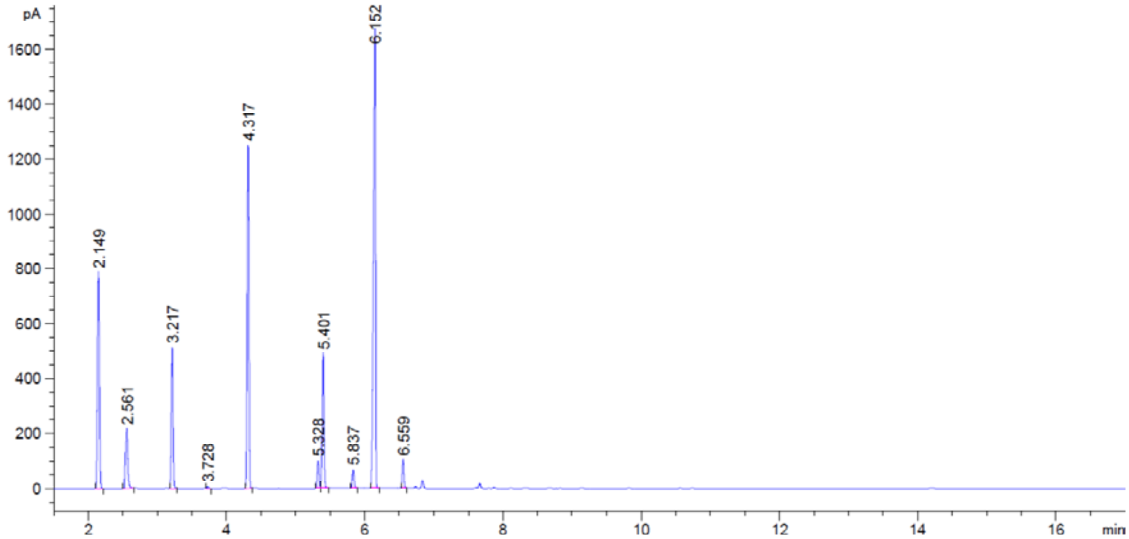

The GC-FID of Table 3 entry 10:

Retention time (min)	2.145	3.217	4.317	5.401	6.152	6.559	8.661
Chemical	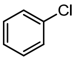	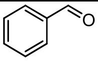	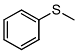	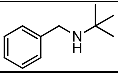	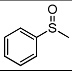	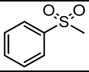	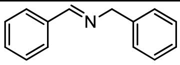
